# Impaired TRPV4-eNOS signaling in trabecular meshwork elevates intraocular pressure in glaucoma

**DOI:** 10.1073/pnas.2022461118

**Published:** 2021-04-14

**Authors:** Pinkal D. Patel, Yen-Lin Chen, Ramesh B. Kasetti, Prabhavathi Maddineni, William Mayhew, J. Cameron Millar, Dorette Z. Ellis, Swapnil K. Sonkusare, Gulab S. Zode

**Affiliations:** ^a^Department of Pharmacology and Neuroscience, North Texas Eye Research Institute, University of North Texas Health Science Center at Fort Worth, Fort Worth, TX 76107;; ^b^Robert M. Berne Cardiovascular Research Center, University of Virginia, Charlottesville, VA 22908;; ^c^Department of Pharmaceutical Sciences, North Texas Eye Research Institute, University of North Texas Health Science Center at Fort Worth, Fort Worth, TX 76107;; ^d^Molecular Physiology and Biological Physics, University of Virginia School of Medicine, Charlottesville, VA 22908

**Keywords:** glaucoma, trabecular meshwork, TRPV4, nitric oxide, fluid flow

## Abstract

Primary Open Angle Glaucoma (POAG) is the most common form of glaucoma that leads to irreversible vision loss. Dysfunction of trabecular meshwork (TM) tissue, a major regulator of aqueous humor (AH) outflow resistance, is associated with intraocular pressure (IOP) elevation in POAG. However, the underlying pathological mechanisms of TM dysfunction in POAG remain elusive. In this regard, transient receptor potential vanilloid 4 (TRPV4) cation channels are known to be important Ca^2+^ entry pathways in multiple cell types. Here, we provide direct evidence supporting Ca^2+^ entry through TRPV4 channels in human TM cells and show that TRPV4 channels in TM cells can be activated by increased fluid flow/shear stress. TM-specific TRPV4 channel knockout in mice elevated IOP, supporting a crucial role for TRPV4 channels in IOP regulation. Pharmacological activation of TRPV4 channels in mouse eyes also improved AH outflow facility and lowered IOP. Importantly, TRPV4 channels activated endothelial nitric oxide synthase (eNOS) in TM cells, and loss of eNOS abrogated TRPV4-induced lowering of IOP. Remarkably, TRPV4-eNOS signaling was significantly more pronounced in TM cells compared to Schlemm’s canal cells. Furthermore, glaucomatous human TM cells show impaired activity of TRPV4 channels and disrupted TRPV4-eNOS signaling. Flow/shear stress activation of TRPV4 channels and subsequent NO release were also impaired in glaucomatous primary human TM cells. Together, our studies demonstrate a central role for TRPV4-eNOS signaling in IOP regulation. Our results also provide evidence that impaired TRPV4 channel activity in TM cells contributes to TM dysfunction and elevated IOP in glaucoma.

## Significance

Primary Open Angle Glaucoma (POAG) is the most common form of glaucoma that leads to irreversible vision loss and blindness worldwide. POAG is often associated with elevated intraocular pressure (IOP). The trabecular meshwork (TM), a molecular sieve-like structure, tightly controls IOP by constantly adjusting the resistance to aqueous humor (AH) outflow. In POAG, there is increased resistance to AH outflow, elevating IOP. Here, we show that mechanically activated TRPV4 channels in TM play a critical role in IOP regulation via calcium-mediated activation of eNOS signaling and production of nitric oxide (NO). Importantly, we show that impaired TRPV4 channel activity results in reduced NO bioavailability and elevated IOP in glaucoma. Our findings support impaired TRPV4-eNOS signaling to the pathogenesis of POAG.

Glaucoma is a heterogenic group of multifactorial neurodegenerative diseases characterized by progressive optic neuropathy. It is the leading cause of irreversible vision loss with more than 70 million people affected worldwide (1), and the prevalence is estimated to increase to 111.6 million by the year 2040 (2). Primary open angle glaucoma (POAG) is the most common form of glaucoma, accounting for ∼70% of all cases (1). POAG is characterized by progressive loss of retinal ganglion cell axons that leads to an irreversible loss of vision (1, 3). Elevated intraocular pressure (IOP) is a major, and the only treatable, risk factor associated with POAG (4). The trabecular meshwork (TM), a molecular sieve-like structure, maintains homeostatic control over IOP by constantly adjusting the resistance to aqueous humor (AH) outflow. In POAG, there is increased resistance to AH outflow, elevating IOP (5). This increase in AH outflow resistance is associated with dysfunction of the TM (6–8).

The TM has an intrinsic ability to sense the AH flow and regulate outflow facility to maintain IOP homeostasis (6), although the precise flow-sensing mechanisms in TM cells are unclear. In this regard, transient receptor potential vanilloid 4 (TRPV4) cation channels have emerged as a major flow-activated Ca^2+^ entry pathway in multiple cell types (9–12). Upon activation, TRPV4 channels allow localized Ca^2+^ influx (termed as TRPV4 sparklets), which influences a variety of cellular homeostatic processes (13, 14). TRPV4 sparklets are spatially restricted signals with a spatial spread (maximum width at half maximal amplitude) of ∼11 microns (13). Treatment with a selective TRPV4 channel activator GSK1016790A (GSK101) lowered IOP in rats and mice (15). Furthermore, baseline IOP was higher in global TRPV4^−/−^ mice compared to their wild-type (WT) littermates (15). However, the exact cell type responsible for these IOP-lowering effects is not known. Previous studies have shown that TRPV4 channel protein is expressed in TM cells and tissues (15, 16). The physiological roles of TRPV4 channels in TM cells (TRPV4_TM_) and downstream signaling mechanisms remain unknown. TM constitutively expresses Ca^2+^-sensitive endothelial nitric oxide synthase (eNOS) (17), a known regulator of outflow facility and IOP (18–22). In vascular endothelial cells, TRPV4 channels are important regulators of eNOS activity (23–26). We, therefore, hypothesized that TRPV4_TM_-eNOS signaling promotes outflow facility and reduces IOP.

Glaucoma-associated pathological changes are known to impair physiological function of TM (8). One of the hallmarks of the glaucomatous TM is its inability to maintain normal IOP and AH outflow resistance (6). Here, we postulated that impaired TRPV4_TM_-eNOS signaling contributes to TM dysfunction and elevated IOP in glaucoma. In this report, our studies in human TM cells and TM tissue showed shear stress–mediated activation of TRPV4-eNOS signaling. Moreover, reduced AH outflow and elevated IOPs were observed in TM-specific TRPV4^−/−^ (TRPV4_TM_^−/−^) mice and eNOS^−/−^ mice. Importantly, TRPV4_TM_ activity and shear stress–mediated activation of TRPV4_TM_-eNOS signaling are compromised in human glaucomatous TM cells. Our results provide direct evidence for a physiological role of TRPV4_TM_-eNOS signaling and indicate that impaired TRPV4_TM_-eNOS signaling may underlie TM dysfunction and IOP dysregulation in glaucoma.

## Results

### Presence of Functional TRPV4 Channels in Human Primary TM Cells and Tissues.

**Fig. 1.**
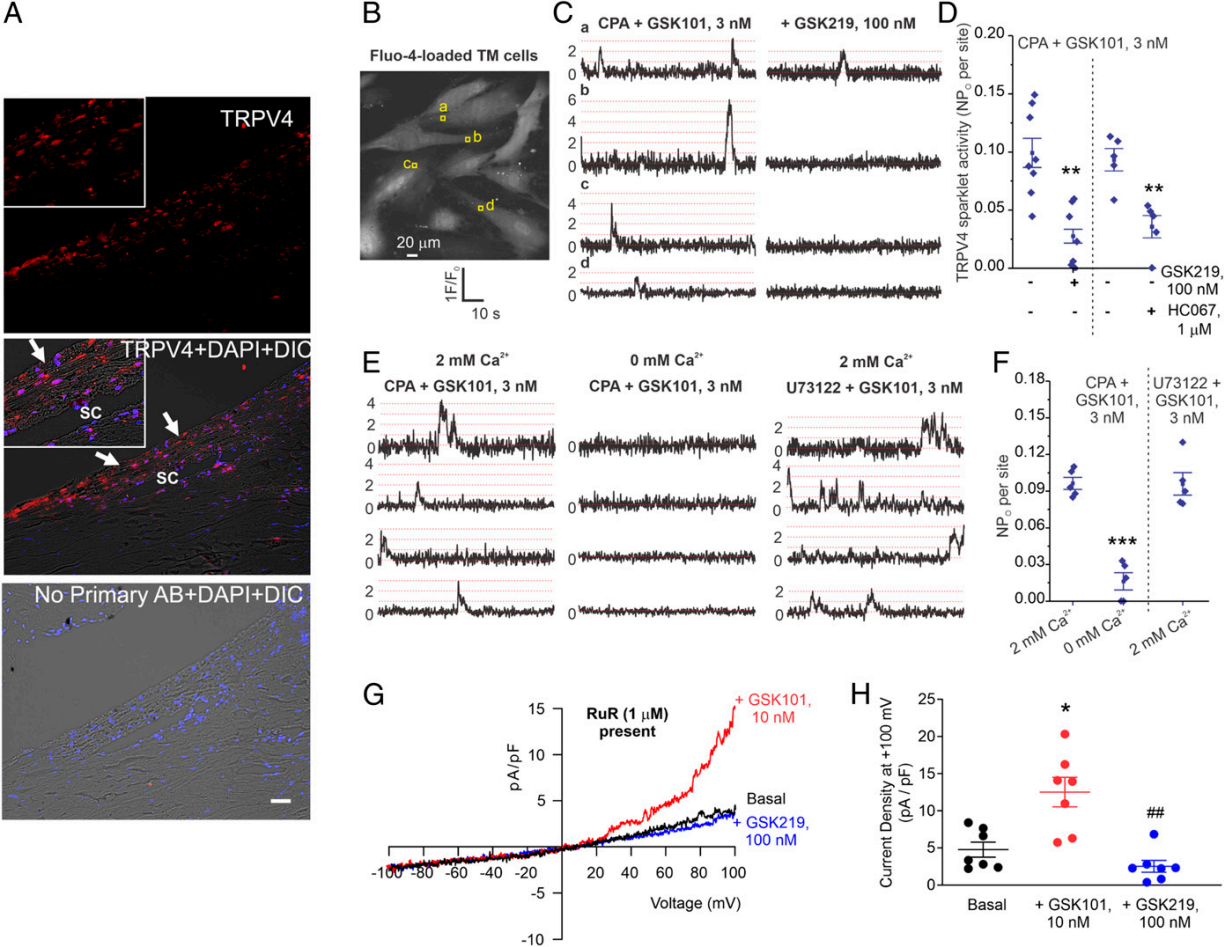
Expression of functional TRPV4 channels in human TM. (*A*) Immunohistochemical images showing expression of TRPV4 (red) in TM and SC endothelium of human donor eyes. No primary antibody control (*Bottom*). Inserts at the left corner of images show the blown out region of TM and SC. *n* = 5 different donors. (Scale bar, 50 μm.) (*B*) TRPV4 Ca^2+^ sparklets represent Ca^2+^ influx through TRPV4 channels on TM cell membranes. Grayscale image of a field of view with ∼10 TM cells; square boxes are the identifiers that represent the pixels with peak amplitudes at the TRPV4 sparklet sites. Experiments were performed in the presence of CPA (sarcoplasmic Ca^2+^-ATPase inhibitor, 20 μM) and GSK1016790A (GSK101, TRPV4 activator, 3 nM). (*C*) Representative F/F_0_ traces generated using the ROIs shown in *B* indicate GSK101-elicited TRPV4 sparklets. Dotted red lines represent quantal level or single-channel amplitude (0.29 ΔF/F_0_). (*D*) Averaged TRPV4 sparklet activity before or after the addition of TRPV4 inhibitor GSK219 (100 nM, *n* = 7 to 8) or HC067047 (HC067, 1 μM). *n* = 5 to 8, **P < 0.01 versus GSK101 (3 nM). TRPV4 sparklet activity is expressed as NP_O_ per site. N represents the number of TRPV4 channels at a site, and P_O_ is the open state probability of the channels. Representative F/F_0_ traces show TRPV4 sparklet activity at four different sites from a field of view (*E*) and averaged TRPV4 sparklet activity (*F*). Experiments were performed in the presence of CPA (20 μM) or U73122 (phospholipase inhibitor, 3 μM). (*G*) Representative traces for ionic currents through TRPV4 channels in TM cells under baseline condition and in the presence of GSK101 (10 nM), recorded in the whole-cell patch configuration. Experiments were performed in the presence of RuR (1 μM) to block Ca^2+^ entry at negative voltages. (*H*) Averaged outward currents in TM cells at +100 mV under basal conditions and in the presence of GSK101 (10 nM) or GSK101 + GSK219 (100 nM). *n* = 7, *P < 0.05 versus Basal, and ^##^*P* < 0.05 versus GSK219. Data are presented as mean ± SEM.

We first examined the localization of TRPV4 channel protein in TM tissue by immunostaining postmortem human donor eyes. TRPV4 channel protein was found in the TM tissue and Schlemm’s canal (SC) cells (Fig. 1*A*). Immunostaining of mouse eyes with TRPV4 and alpha-smooth muscle actin (α-SMA, a marker of TM) antibodies revealed that TRPV4 protein is localized to mouse TM tissue (*SI Appendix*, Fig. S1). Next, we examined TRPV4 channel activity in human primary TM cells (*n* = 4 donor strains) using high-speed Ca^2+^ imaging and patch clamp analysis. Ca^2+^ influx signals through individual TRPV4_TM_ channels [“TRPV4 sparklets” (13)] were recorded in fluo-4 acetoxymethyl (AM)-loaded TM cells using spinning disk confocal microscopy (Fig. 1*B*). A 1.6 μm^2^ region of interest (ROI) was placed at each sparklet site to generate the fractional fluorescence (F/F_0_) traces (Fig. 1*C*). The quantal level, or single-channel amplitude, was ΔF/F0 of 0.29, similar to what we have reported in vascular cells (13). The activity of TRPV4_TM_ sparklets was increased by TRPV4 channel activator, GSK101 (3 nM), and inhibited by TRPV4 inhibitors GSK2193874 (GSK219, 100 nM, Fig. 1*D*) or HC067047 (HC067, 1 μM). The phospholipase C inhibitor, U73122 (3 μM), did not alter TRPV4_TM_ sparklet activity (Fig. 1 *E* and *F*). Furthermore, replacing 2 mM extracellular physiological solution with a 0 mM Ca^2+^ solution quickly abolished the activity of TRPV4_TM_ sparklets (Fig. 1 *E* and *F*), indicating that TRPV4_TM_ sparklets were Ca^2+^ influx signals. GSK101 also elicited robust ionic currents in TM cells that were abolished by GSK219 (Fig. 1 *G* and *H*). These results firmly establish the presence of functional TRPV4 channels and Ca^2+^ influx through these channels in TM cells.

### TRPV4 Channel Increases Outflow Facility and Lowers IOP in Mice.

**Fig. 2.**
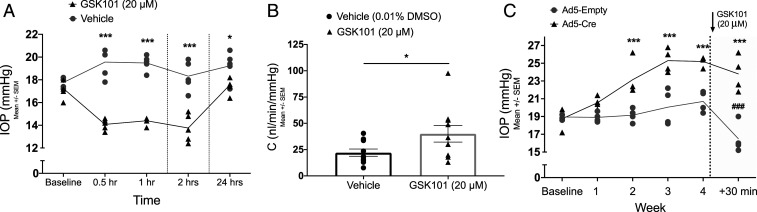
TRPV4 channels regulate outflow facility and IOP. (*A*) TRPV4 activation lowers IOP in C57BL/6J mice. Following measurement of dark-adapted baseline IOP, animals were topically administered 5 μL eyedrops of 20 μM GSK101 in one eye and 0.01% DMSO vehicle in the contralateral eye. Dark-adapted IOP was measured at 0.5, 1, 2, and 24 h intervals posttreatment. Data are represented as mean ± SEM; ***P < 0.001 versus same time point in vehicle group, *n* = 5 eyes/group; two-way ANOVA followed by Bonferroni’s post hoc test. (*B*) Comparison of conventional outflow facility between GSK101-treated and vehicle-treated mouse eyes. Animals were administered 5 μL eyedrops of 20 μM GSK101 in one eye and 0.01% DMSO in the contralateral eye 30 min prior to the measurement of conventional outflow facility using a constant-flow infusion method. Data are represented as mean ± SEM; *P < 0.05 versus vehicle, *n* = 10 eyes/group; unpaired two-tailed *t* test. (*C*) Elevation of IOP in Ad5-Cre–transduced TRPV4^f/f^ mice. Following baseline measurement of dark-adapted nighttime IOP, animals were injected with Ad5-Cre in one eye and Ad5-Empty in the contralateral control eye. Nighttime IOP was measured at 1, 2, 3, and 4 wk intervals. Following the fourth week of IOP measurement, both eyes were treated with 5 μL GSK101 (20 μM), and nighttime IOP was measured 30 min posttreatment. Data are represented as mean ± SEM; ***P < 0.001 versus same time point in control group (Ad5-Empty), ^###^*P* < 0.001 versus previous time point (4 wk) in same group. *n* = 4 eyes/group; two-way ANOVA followed by Bonferroni’s post hoc test.

Previous studies have reported conflicting results regarding the role of TRPV4 in IOP regulation. Luo et al. demonstrated that activation of TRPV4 channels reduces IOP in rats and mice (15). Another study by Ryskamp et al. demonstrated that inhibiting TRPV4 channels reduces elevated IOP in a microbead occlusion model of glaucoma (16). Unlike previously published reports, we sought to understand the role of TRPV4 channels in regulating the outflow facility and IOP under physiological conditions. Following measurements of baseline nighttime IOP, 3 mo old C57BL/6J mice were treated with 5 μL eyedrops of 20 μM GSK101 in one eye and vehicle (0.01% dimethyl sulfoxide [DMSO]) in the contralateral control eye. IOP was measured at 0.5, 1, 2, and 24 h posttreatment. GSK101 treatment significantly reduced the IOP, a decrease in IOP that started at 0.5 h and lasted until 24 h posttreatment (Fig. 2*A*). No decrease in IOP was observed in the control eyes. Lower concentration of GSK 101 (1 μM) did not alter IOP significantly in C57BL/6J mice (*SI Appendix*, Fig. S2). Next, we determined whether activation of TRPV4 channels improves outflow facility. C57BL/6J mice were treated with 5 μL eyedrops of 20 μM GSK101 or 0.01% DMSO vehicle 30 min prior to measurement of conventional outflow facility using the constant-flow infusion method (27). GSK101 treatment increased outflow facility significantly compared to vehicle-treated controls (Fig. 2*B*). To determine the importance of TRPV4 channels in maintaining physiological IOP, we induced conditional knockout of TRPV4 from TM tissue (TRPV4^−/−^_TM_) by intravitreal injections of adenovirus 5 (Ad5), expressing Cre in TRPV4^f/f^ mice. Intravitreal injections of Ad5 have been shown to exhibit selective tropism for mouse TM (28–30). The contralateral eyes were injected with Ad5-Empty. Weekly nighttime IOP measurements revealed a significant increase of IOP starting at week two posttransduction in Ad5-Cre–injected eyes compared to Ad5-Empty–injected contralateral control eyes (Fig. 2*C*). Next, we examined the TRPV4_TM_-specific nature of a GSK101-induced decrease in IOP. Following week four IOP measurements on TRPV4^f/f^ mice, both eyes were treated with topical eyedrops of 20 μM GSK101, and IOP measurements were performed 30 min posttreatment. A significant decrease in IOP was observed in Ad5-Empty–transduced eyes compared to a slight drop in Ad5-Cre–transduced eyes (Fig. 2*C*). Absence of an IOP-lowering effect in Ad5-Cre–transduced TRPV4^f/f^ mice indicated that GSK101 selectively targets TRPV4_TM_ channels for lowering the IOP. Together, these data support a critical role of TRPV4_TM_ channels in maintaining a low IOP.

### Fluid Flow–Mediated Shear Stress Activates TRPV4 Channels in the TM.

**Fig. 3.**
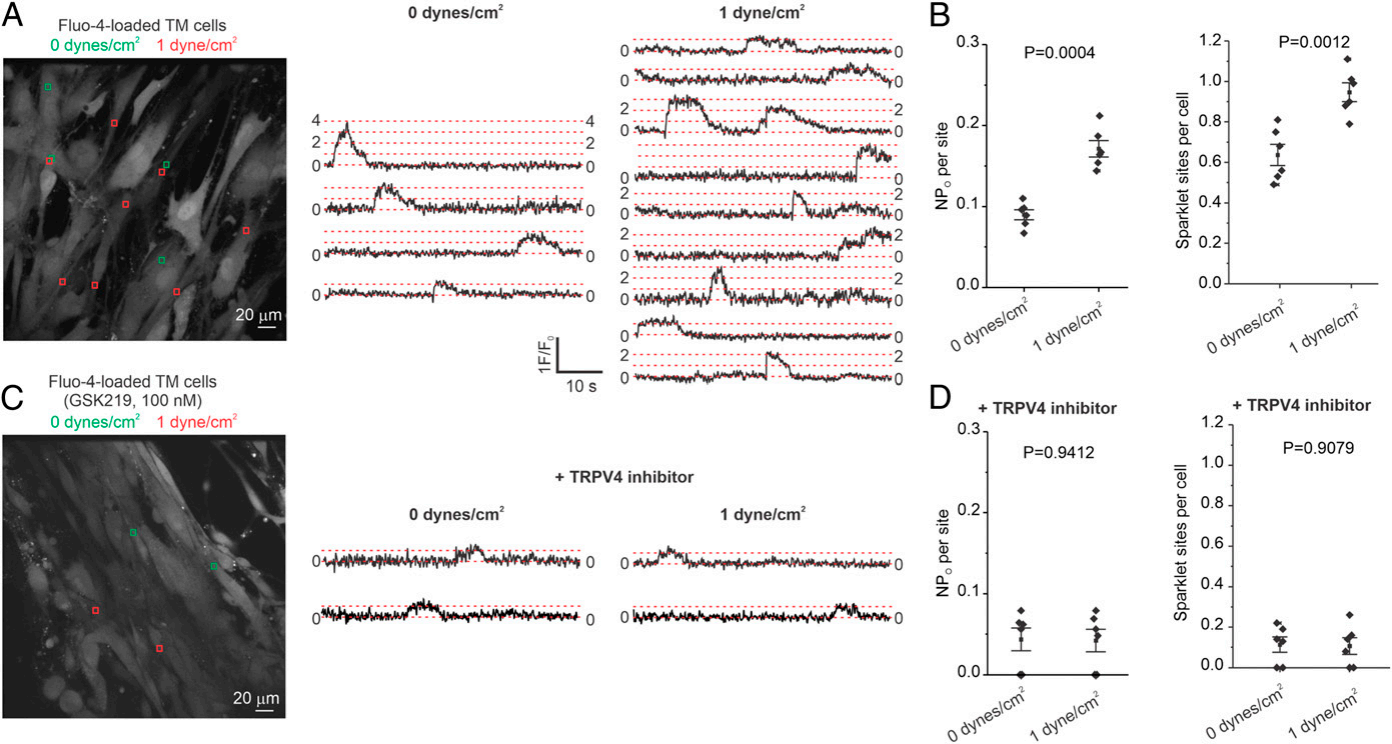
Flow/shear stress increases TRPV4 channel activity in TM cells. (*A*, *Left*) A grayscale image of fluo-4–loaded TM cells; the ROIs represent TRPV4 sparklet sites in the absence of flow (green square boxes; 0 dyne/cm^2^) or the presence of flow/shear stress (red square boxes; 1 dyne/cm^2^); (*A*, *Right*) F/F_0_ traces from the ROIs shown in the grayscale image indicate TRPV4 sparklet activity before (0 dyne/cm^2^) and after (1 dyne/cm^2^) flow/shear stress. (*B*) Averaged TRPV4 sparklet activity (*Left*) or the number of sparklet sites per cell (*Right*) in the absence (0 dyne/cm^2^, *n* = 6) or presence of flow (1 dyne/cm^2^, *n* = 6). (*C*, *Left*) A grayscale image of fluo-4–loaded TM cells in the presence of GSK219 (TRPV4 inhibitor, 100 nM); the ROIs represent TRPV4 sparklet sites in the absence (green square boxes; 0 dyne/cm^2^) and or presence of flow/shear stress (red square boxes; 1 dyne/cm^2^). (*C*, *Right*) F/F_0_ traces from the ROIs on the grayscale image indicate TRPV4 sparklet activity before (0 dyne/cm^2^) and after (1 dyne/cm^2^) flow/shear stress. Experiments were performed in the presence of the TRPV4 channel inhibitor GSK219 (100 nM). (*D*) Averaged TRPV4 sparklet activity (*Left*) or the number of sparklet sites per cell (*Right*) in the absence (0 dyne/cm^2^, *n* = 6) or presence of flow (1 dyne/cm^2^, *n* = 6). Experiments were performed in the presence of the TRPV4 channel inhibitor GSK219. Data are presented as mean ± SEM.

Although direct mechanosensation by TRPV4 channels is contested (31), TRPV4 channels are known to be mechanically activated by increases in shear stress (9, 12, 32). We hypothesized that shear stress activates TRPV4_TM_ channels, thereby promoting TM function and lowering IOP. Shear stress was increased by altering the fluid flow in a rectangular flow chamber. Increasing the shear stress from 0 to 1 dyne/cm^2^ increased the number of TRPV4_TM_ sparklet sites per TM cell and activity per TRPV4_TM_ sparklet site in human primary TM cells (Fig. 3 *A* and *B*). Moreover, in the presence of TRPV4 channel inhibitor GSK219, increased shear stress was unable to activate TRPV4_TM_ sparklets (Fig. 3 *C* and *D*). These results provided direct evidence that increased fluid flow/shear stress promotes Ca^2+^ influx through TRPV4_TM_ channels.

### TRPV4 Sparklets Activate eNOS Signaling in TM Cells.

**Fig. 4.**
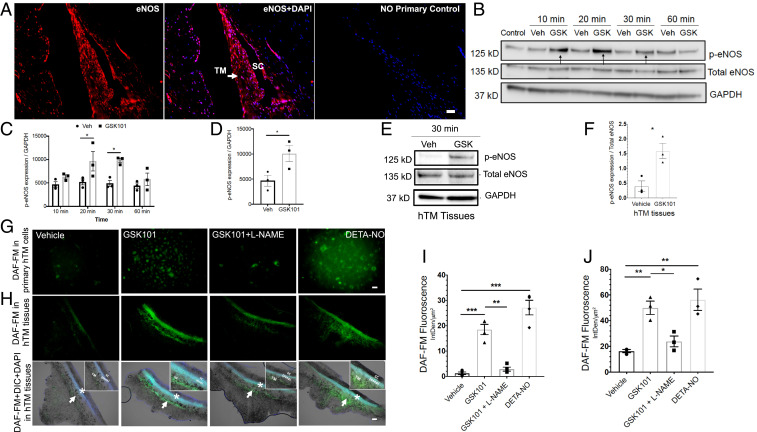
TRPV4 channels are functionally coupled to eNOS in human TM cells and tissues. (*A*) Immunohistochemical images showing expression of eNOS (red) in the human TM and SC endothelium (*n* = 6). Nuclei were counterstained with DAPI (blue; center image). No primary antibody control (*Right*). (Scale bar, 50 μm.) (*B* and *C*) TRPV4 activation leads to phosphorylation of eNOS in TM cells. Representative Western blot image (*B*) and densitometric analysis (*C*) showing expression of p-eNOS, total eNOS, and glyceraldehyde 3-phosphate dehydrogenase (GAPDH) in lysates from cultured transformed TM cells (GTM3) treated with 20 nM GSK101 or 0.001% DMSO vehicle for 10, 20, 30, and 60 min intervals. *P < 0.05 versus vehicle at same time point, *n* = 3/group; unpaired two-tailed *t* test. (*D*) Densitometric analysis of Western blot for p-eNOS levels in primary human TM cells treated with 20 nM GSK101 or 0.001% DMSO vehicle. *P < 0.05 versus vehicle, *n* = 3 donors/group; unpaired two-tailed *t* test. (*E* and *F*) TRPV4-mediated phosphorylation of eNOS in human donor TM tissues. Representative Western blot (*E*) showing expression of p-eNOS, total eNOS, and GAPDH in ex vivo cultured human TM tissues treated with 20 nM GSK101 and 0.01% DMSO vehicle. Densitometric analysis (*F*) compares levels of p-eNOS over eNOS between the groups. *P < 0.05 versus vehicle, *n* = 3/group; unpaired two-tailed *t* test. (*G*–*J*) TRPV4 activation leads to NO production in primary TM cells and ex vivo cultured human TM tissues. Images and comparisons of DAF-FM intensity (IntDen/μm^2^) in normal primary human TM cells (*G* and *I*) and ex vivo cultured human TM tissues (*H* and *J*) treated with 0.001% DMSO vehicle, 20 nM GSK101, 20 nm GSK101 + 100 μM l-NAME, or 100 μM DETA/NO. Arrows indicate TM, and * indicates SC region (*H*). Insets on the right corner of bottom images show blown out regions of TM and SC. (Scale bar, 50 μm for *H* and 100 μm for *J*.) *P < 0.5; **P < 0.01; ***P < 0.001, *n* = 3 cell strains/group; one-way ANOVA followed by Bonferroni’s post hoc test.

Previous studies suggest an important role for eNOS in human TM and SC cells (18, 19, 33). Moreover, shear stress has been shown to increase eNOS phosphorylation and endogenous NO production in outflow pathway cells (34). Studies in the vasculature demonstrated a clear link between TRPV4-eNOS signaling and vasodilation (23–26). We therefore hypothesized that TRPV4_TM_ channels reduce IOP and improve outflow facility via activation of eNOS. Age-matched paraffin-fixed human anterior segment tissues (*n* = 6 donors) that were stained with eNOS antibody showed robust eNOS labeling in TM and SC cells (Fig. 4*A*). We further explored whether eNOS and TRPV4 is present in primary human TM cells and SC cells. Western blot and its densitometric analysis demonstrated that both primary TM and SC cells exhibit similar levels of TRPV4 and eNOS protein levels (*SI Appendix*, Fig. S3). We next sought to examine whether TRPV4 channel activator GSK101 promotes eNOS activity in human TM cells and TM tissues (Fig. 4 *B*–*F*). GTM3 cells were treated with either vehicle or GSK101 for various time periods, and cellular lysates were examined by Western blot analysis (Fig. 4 *B* and *C*). Treatment with GSK101 led to a significant increase in eNOS phosphorylation levels starting at 10 min posttreatment. Consistent with this, GSK101 significantly induced eNOS phosphorylation in primary human TM cells (Fig. 4*D* and *SI Appendix*, Fig. S4). Furthermore, GSK101 treatment induced phosphorylation of eNOS in human TM tissues in an ex vivo cultured corneoscleral segment model (35) (Fig. 4 *E* and *F*). Human cultured corneoscleral segments were treated with GSK101 or vehicle for 30 min. Densitometric analysis of Western blots revealed significantly increased levels of phosphorylated eNOS p-eNOS after treatment with GSK101. We next examined whether activation of TRPV4 channels increases nitric oxide (NO) production using fluorescent NO indicator, 4-amino-5 methylamino-2′,7′-difluorofluorescenin diacetate (DAF-FM) staining. Human TM cells (Fig. 4 *G* and *I*) and ex vivo cultured human corneoscleral segment tissues (Fig. 4 *H* and *J*) were pretreated with DAF-FM and then subsequently treated with 20 nM GSK101 with or without l-NAME (N(G)-Nitro-L-arginine methyl ester; a pan-nitric oxide synthase inhibitor and negative control) or diethylenetriamine NONOate (DETA-NONOate) (NO donor; positive control). Treatment with GSK101 significantly increased NO production in TM cells and tissues as evident from increased DAF-FM fluorescence intensity, which was partially blocked by pan-NOS inhibitor. The positive control, DETA-NONOate, also significantly increased NO production in primary human TM cells.

### TRPV4-eNOS Coupling Is More Pronounced in TM Cells Compared to SC Cells.

**Fig. 5.**
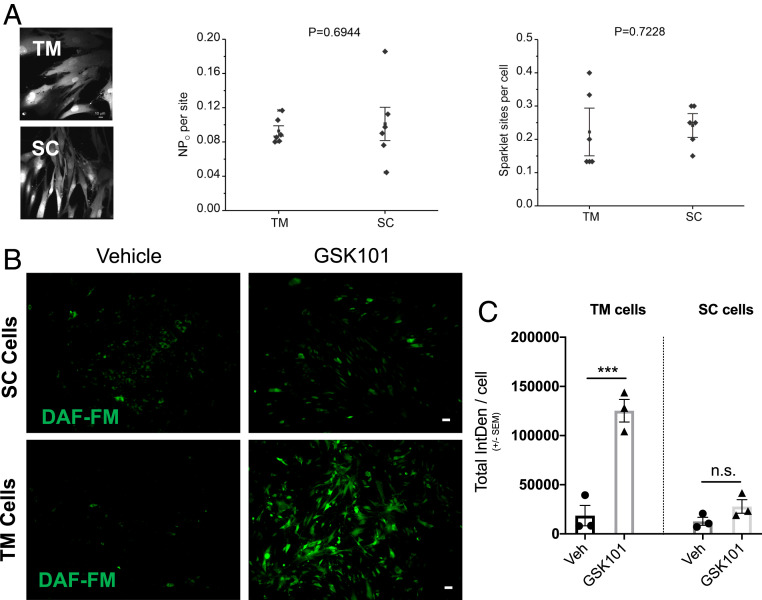
Comparison of TRPV4 sparklet channel activity between primary human TM and SC cells. (*A*) TRPV4-mediated changes in sparklet activity in TM and SC cells. (*Left*) Grayscale images of fluo-4–loaded TM and SC cells. Averaged TRPV4 sparklet activity (center) and the number of sparklet sites per cell (*Right*) in TM and SC cells after treatment with TRPV4 agonist GSK101 (3 nM). (*B* and *C*) Comparison of TRPV4 channel–mediated increase in NO production in TM and SC cells. Representative images (*B*) and analysis (*C*) of DAF-FM intensity in normal primary human TM and SC cells treated with 0.001% DMSO vehicle or 20 nM GSK101. (Scale bar, 50 μm.) ***P < 0.001, *n* = 3 cell strains/group; one-way ANOVA followed by Bonferroni’s post hoc test. n.s., not significant.

Given that TRPV4 and eNOS is expressed in both TM and the SC (36), we compared the functional coupling of TRPV4-eNOS in primary human TM and SC cells. Pharmacological activation of TRPV4 channels with agonist GSK101 (3 nM) resulted in similar sparklet activity levels in both TM (*n* = 3) and SC (*n* = 3) cells (Fig. 5*A*). We then compared levels of TRPV4-mediated NO production in TM and SC cells using DAF-FM assay for NO detection. Studies in human primary TM cells (*n* = 3 strains) and SC cells (*n* = 3 strains) demonstrated that GSK101-induced increase in NO production is higher in TM cells than in SC cells (Fig. 5 *B* and *C*).

### TRPV4_TM_-eNOS Signaling Lowers IOP.

**Fig. 6.**
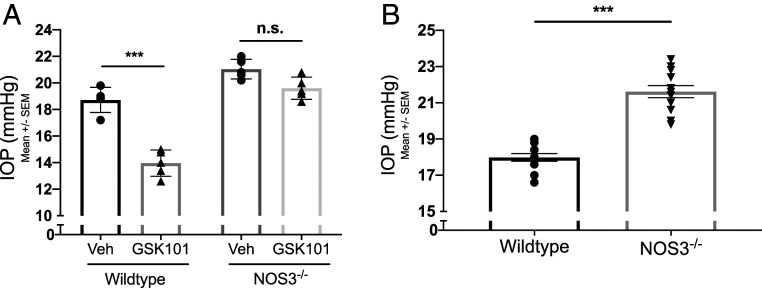
TRPV4-mediated lowering of IOP is eNOS dependent. (*A*) Effect of TRPV4 activation on IOP in WT and NOS3^−/−^ mice. WT and NOS3^−/−^ animals were administered 5 μL eyedrops of 20 μM GSK101 in one eye and 0.01% DMSO in the contralateral eye 30 min prior to the measurement of dark-adapted IOP. Data are represented as mean ± SEM; ***P < 0.001 versus vehicle group, *n* = 5 eyes/group; one-way ANOVA followed by Bonferroni’s post hoc test. (*B*) Dark-adapted baseline IOP in WT C57BL/6J mice and NOS3^−/−^ mice. Data are represented as mean ± SEM; ***P < 0.001 versus WT, *n* = 12 eyes/group; unpaired two-tailed *t* test. n.s., not significant.

eNOS-derived NO is an essential regulator of IOP homeostasis (18, 19, 37, 38). We utilized NOS3^−/−^ mice to determine whether TRPV4-mediated lowering of IOP is dependent on eNOS signaling. WT and NOS3^−/−^ mice were treated with 20 μm GSK101 in one eye and 0.01% DMSO vehicle in the contralateral control eye 30 min prior to nighttime IOP measurements. GSK101 treatment significantly lowered IOP in WT mice but not in NOS3^−/−^ mice (Fig. 6*A*). Consistent with these results, nighttime IOPs were significantly higher in global NOS3^−/−^ mice compared to age-matched WT littermates (Fig. 6*B*). These data suggest that eNOS is necessary for TRPV4-mediated lowering of IOP.

### TRPV4 Channel Activity Is Impaired in Glaucoma.

**Fig. 7.**
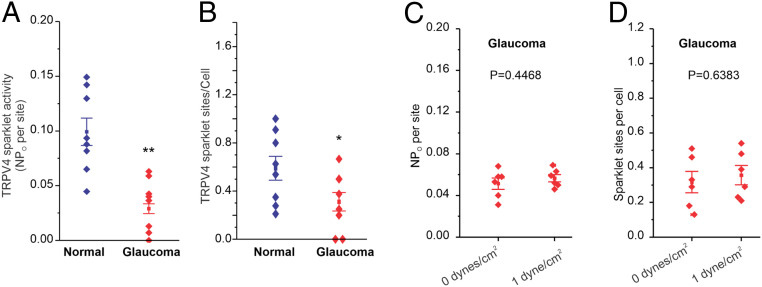
Activity of TRPV4 channels is impaired in glaucomatous TM cells. (*A* and *B*) Averaged TRPV4 sparklet activity in normal (*n* = 8) and glaucomatous (*n* = 8) primary human TM cells treated with 3 nM TRPV4 agonist GSK101. TRPV4 sparklet activity is expressed as NP_O_ per site and number of sparklet sites per cell. N represents the number of channels at a site, and P_O_ is the open state probability of the channels. Data are presented as mean ± SEM. (*C* and *D*) Sensitivity to shear stress is diminished in glaucoma. Averaged TRPV4 sparklet activity in the absence (0 dyne/cm^2^, *n* = 6) or presence (1 dyne/cm^2^, *n* = 6) of flow/shear stress. The experiments were performed in the presence of GSK101 (3 nM). Data are presented as mean ± SEM. *P <0.05, **P < 0.01 vs normal; unpaired two-tailed *t* test.

Although TM can sense the changes in fluid flow and regulate IOP under normal conditions, pathological mechanisms for the failure of TM to maintain normal IOP in glaucoma remain elusive. We hypothesized that impaired TRPV4_TM_-eNOS signaling contributes to IOP elevation in glaucoma. TRPV4_TM_ sparklet activity was assessed in normal and glaucomatous primary human TM cells (Fig. 7 *A* and *B* and *SI Appendix*, Fig. S5). TRPV4 sparklet activity per site and the number of sparklet sites per cell were significantly lower in TM cells from glaucoma patients. While an increase in shear stress from 0 dynes/cm^2^ to 1 dyne/cm^2^ elevated TRPV4_TM_ sparklet activity in normal TM cells (Fig. 3), it was unable to increase TRPV4_TM_ sparklet activity in glaucomatous TM cells (Fig. 7 *C* and *D*), further supporting impaired TRPV4_TM_ channel regulation in glaucoma. We next examined the possibility that lower TRPV4_TM_ activity is a result of reduced expression levels (*SI Appendix*, Fig. S6). Western blot analysis demonstrated slightly increased TRPV4 protein levels in glaucomatous TM cells, indicating that impaired channel regulation, rather than channel expression, contributes to the lowering of TRPV4 channel activity in glaucoma.

### TRPV4-Mediated Shear Stress Transduction Is Compromised in Glaucoma.

**Fig. 8.**
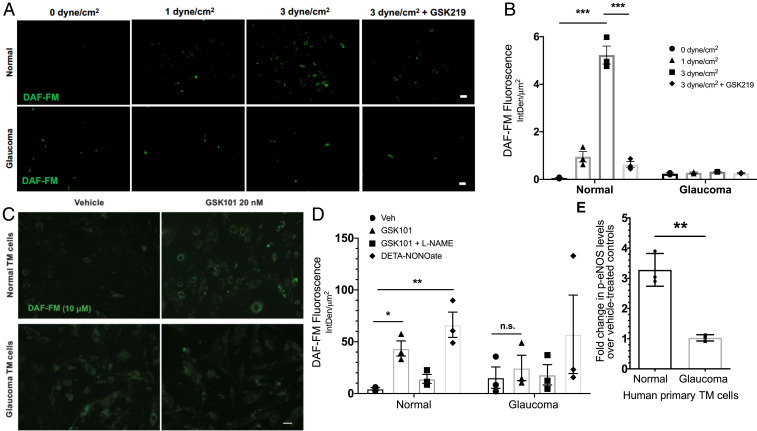
(*A* and *B*) Shear stress–mediated TRPV4-eNOS signaling is diminished in glaucomatous TM cells. (*A*) Normal and glaucomatous primary human TM cells were subjected to different shear stress conditions (0, 1, and 3 dyne/cm^2^) and treated with NO-binding DAF-FM dye to determine shear stress–mediated NO production. TRPV4 antagonist GSK219 (100 nM) was used to determine the role of TRPV4 in shear stress transduction. High shear stress (3 dyne/cm^2^) led to increased DAF-FM fluorescence intensity, which was reduced by TRPV4 antagonist GSK219. *n* = 3 normal and 2 glaucoma. (Scale bar, 50 μM.) (*B*) Mean DAF-FM fluorescence intensity/μm^2^ in normal and glaucomatous primary TM cells. ***P < 0.001, *n* = 3 donor strains per group; one-way ANOVA followed by Bonferroni’s post hoc test. (*C*–*E*) TRPV4-eNOS coupling is impaired in glaucomatous TM cells. (*C*) Representative image comparing TRPV4-mediated NO production in normal and glaucomatous primary human TM cells using DAF-FM assay. Normal and glaucomatous primary TM cells were pretreated with NO-binding DAF-FM fluorescent dye (green) and then treated with 0.001% DMSO vehicle, 20 nM GSK101, 20 nm GSK101 + 100 μM l-NAME, or 100 μM DETA/NO. *n* = 3/group (scale bar, 50 μm). (*D*) Quantification of DAF-FM fluorescence intensity/μm^2^ in normal and glaucomatous primary TM cells. *P < 0.05, **P < 0.01 versus the vehicle-treated group, *n* = 3 cell strains/group; one-way ANOVA followed by Bonferroni’s post hoc test. (*E*) Densitometric analysis of Western blot for p-eNOS showing relative fold change in p-eNOS levels in normal and glaucomatous primary human TM cells over their respective vehicle-treated controls. *n* = 3 cell strains/group; unpaired two-tailed *t* test. n.s., not significant.

Although direct mechanosensitive nature of TRPV4 channels is debatable, TRPV4 channels are widely known to be activated by flow/shear stress (9, 11, 24). Shear stress also increases eNOS phosphorylation and endogenous NO production in outflow pathway cells (34). Therefore, we examined the possibility that shear stress–induced NO production is reduced in glaucomatous primary human TM cells. Shear stress increased NO production in normal TM cells (1 to 3 dyne/cm^2^) in a TRPV4 channel–dependent manner (Fig. 8 *A* and *B*). Importantly, shear stress was unable to increase NO levels in glaucomatous TM cells, data that were consistent with impaired TRPV4_TM_ channel activity in glaucoma.

### TRPV4-eNOS Coupling Is Disrupted in Glaucoma.

We further tested the hypothesis that impaired TRPV4_TM_ channel activity in glaucoma results in reduced eNOS signaling and NO production. DAF-FM assay for measurements of intracellular NO levels revealed that GSK101 significantly increased NO production in primary TM cells from normal donor but not in glaucomatous TM cells (Fig. 8 *C* and *D*). This was corroborated by Western blot analysis of p-eNOS (Fig. 8*E* and *SI Appendix*, Fig. S7), which revealed a threefold increase in eNOS phosphorylation in GSK101-treated normal TM cells over vehicle-treated normal TM cells. In glaucomatous TM cells, however, GSK101 was unable to increase eNOS phosphorylation.

## Discussion

In this study, we assessed the role of TRPV4_TM_ channels in flow-sensing signaling mechanisms under normal conditions and its impairment in glaucoma. We show that fluid flow–induced shear stress activates TRPV4_TM_ channels and induces eNOS-mediated NO production. Furthermore, we demonstrate that the activity of TRPV4_TM_ channels is impaired in glaucoma, rendering TM cells insensitive to fluid flow–induced shear stress. Glaucoma-associated functional impairment of TRPV4_TM_ channels may contribute to IOP elevation over time, leading to glaucomatous neurodegeneration.

In perfusion-cultured human anterior segments, Bradley et al. demonstrated that conventional outflow resistance is actively regulated in order to maintain normal IOP (39). In this context, any potential mechanism for IOP regulation would require a means to sense the magnitude of local physical forces, including flow-induced shear stress and stretch. Based on the previous observation of adaptive responses in the outflow tissues (6, 39, 40), we postulated that mechanotransduction of physical forces likely occurs within the TM and SC. Mechanotransduction at the level of TM is essential for maintaining normal IOP. TM cells are continuously exposed to the flow of AH and are therefore subjected to an array of dynamic physical forces (41, 42). A recent study has demonstrated TM cells respond to 0.5 dyne/cm^2^ shear stress by increasing Ca^2+^ signals (43). In literature, there is little evidence to suggest how much shear stress TM cells experience in vivo, and it is likely that pressure differentials are a critical determinant of TM response. It is also likely that TM cells undergo low shear stress compared to SC cells (41, 42). In vascular endothelium, TRPV4 channels are known to be activated by flow-induced shear stress (9, 11, 24). Here, we demonstrate that shear stress sensation by TM cells involves activation of TRPV4_TM_ channels, leading to transient and localized Ca^2+^ entry events into TM cells termed “TRPV4 sparklets.” This effect was reversed by selective TRPV4 channel antagonist GSK219, indicating an important role of TRPV4_TM_ channels in shear stress transduction (Figs. 3 *A*–*D* and 8 *A* and *B*). Our studies demonstrate that TM cells have functional TRPV4 channels, and they respond to physiological stimuli by increasing downstream Ca^2+^ responses (Figs. 3*B* and 8*B*). Pathological changes at the TM are known to accumulate overtime and contribute to TM dysfunction and, as a result, affect several physiological processes, including IOP homeostasis (8). We further demonstrate that glaucomatous TM cells derived from POAG donors are insensitive to shear (Fig. 7 *C* and *D*), indicating that TRPV4 channel–mediated mechanotransduction is impaired in glaucomatous primary TM cells.

TRPV4 channels are expressed in several different ocular tissues, including the TM, ciliary body, retina, and cornea. In the TM, these channels have been implicated for their role in IOP regulation (15, 16). We show that topical application of TRPV4 channel activator lowers IOP and improves outflow facility (Fig. 2 *A* and *B*). These results are in agreement with previous reports exploring the effects of TRPV4 activation in mouse eyes (15, 44). To show the importance of TRPV4 channels in maintenance of normal IOP, we selectively deleted TRPV4 channel from mouse TM by intravitreally injecting Ad5-Cre in TRPV4^f/f^ mouse eyes. Selective deletion of TRPV4 channel from the TM using Ad5-Cre vector resulted in IOP elevation, which was more pronounced than the IOP elevation previously reported in the global TRPV4^−/−^ mouse model (15). These data add to the mounting evidence supporting a prominent role for TRPV4 channels in physiological regulation of IOP and outflow facility. We further show that pharmacological activation of TRPV4 channels leads to influx of extracellular calcium, detected as TRPV4 sparklets in real-time using high-speed Ca^2+^ imaging. Although global calcium influx for an extended period of time is considered toxic for cells, our high-speed confocal imaging reveals that TRPV4 sparklets are highly localized and occur over a very short period of time. Furthermore, TM cells have a high tolerance for Ca^2+^, an important second messenger necessary for numerous cellular processes, including cytoskeletal remodeling and cell volume regulation (45).

Our results reveal that TRPV4_TM_ channels are important regulators of eNOS activity and NO levels in TM cells. Pharmacological activation of TRPV4 channels via selective agonist GSK101 leads to increased eNOS activity and production of NO in TM cells. Furthermore, the application of mechanical shear stress also leads to an increase in NO production, which is diminished by inhibiting TRPV4 channels. We further examined the TRPV4-eNOS signaling axis by using the NOS3^−/−^ mouse model. Activation of TRPV4 channels in NOS3^−/−^ mice did not result in a reduction in IOP as observed in WT mice (Fig. 6*A*). This indicates an important role for TRPV4-eNOS signaling in IOP regulation. NO is a known modulator of numerous physiological processes of the eye, including IOP and ocular blood flow (33, 46, 47). In the TM cells, NO has been shown to modulate cell morphology, leading to relaxation of TM (20, 22, 48). Furthermore, treatment with exogenous NO donor compounds has been reported to reduce IOP and increase outflow facility in mice (19), rabbit (21), nonhuman primates (22), and human ex vivo models (37). Despite the apparent therapeutic benefits of exogenous NO, it is important to acknowledge the paradoxical effects of excess NO that may lead to pathological outcomes. At lower concentrations, NO is an important homeostatic mediator that is beneficial for cell survival, whereas at high concentrations, excess NO can lead to nitrosative stress and physiological dysregulation (49). Given the importance of NO in glaucoma, very little is known about the upstream mechanisms that regulate endogenous NO production in the eye.

In the conventional outflow pathway, studies have demonstrated that TM cells respond to mechanical stressors like IOP elevation by altering their morphology (50, 51). A recent study has shown that opening of mechanically activated TRPV4 channels in TM cells via GSK101 leads to Ca^2+^-mediated rearrangement of cytoskeletal actin filaments, which causes cellular contraction and an increase in AH drainage, presumably via the paracellular pathway (44). However, the temporal nature and magnitude of such cellular contractions that lead to increased AH drainage are yet to be determined. NO is known to reverse such contractile changes to TM cell morphology. In this study, we provide a mechanistic insight into a pathway involved in mitigating biomechanical stress-dependent changes by regulating endogenous production of NO. The NOS isoform eNOS is considered to be a major contributor to the NO biosynthesis in the TM, and increased eNOS levels in the mouse TM have been associated with reduction of IOP (18). In contrast to a previous study (52), we observed robust expression of eNOS in human TM tissues obtained from donor eyes. eNOS has also been shown to be expressed in the SC endothelium. As a result, we characterized and compared the TRPV4-eNOS coupling effect in primary human TM and SC cells. We observed no difference in TRPV4 sparklet activity in response to GSK101 treatment between TM and SC cells (Fig. 5*A*). However, TRPV4-mediated increase in NO production was significantly higher in TM cells compared to SC cells (Fig. 5 *B* and *C*). This evidence, in conjunction with our finding that although both TM and SC cells express TRPV4 and eNOS, TM cells possess the α-SMA that is known to regulate cellular morphology. Furthermore, our data show that TM cells show better TRPV4-eNOS coupling compared to SC cells. We postulate that the opening of mechanically activated TRPV4 channels causes transient Ca^2+^ influx, which perhaps results in the contraction-mediated increase in aqueous drainage. Temporally separated, the opening of TRPV4 channels also leads to Ca^2+^-mediated activation of eNOS and NO production, thereby inhibiting the cellular contractile machinery and improving the TM tone. We surmise that such oscillatory contraction and relaxation of TM cells in response to mechanical stress are essential in maintaining normal IOP. Future studies will focus on evaluating the oscillatory nature of this pathway.

Down-regulation of eNOS activity and a reduced availability of NO is associated with POAG (38, 47, 53). We observed that TRPV4-mediated eNOS activity is diminished in glaucomatous primary TM cells when compared to normal TM cells (Fig. 8). Furthermore, pharmacological activation of TRPV4 channels via selective agonist did not result in an increase in NO production in glaucomatous human TM cells (Fig. 8 *C* and *D*). Shear stress–mediated production of endogenous NO was also reduced in glaucomatous primary human TM cells (Fig. 8 *A* and *B*).

In conclusion, our data substantiates the role of TRPV4 channels in shear stress sensation at the TM, NO regulation, and physiological regulation of IOP and outflow facility. Upon mechanical activation, these channels intrinsically regulate endogenous NO production in primary human TM cells. We also demonstrate that TRPV4-mediated lowering of IOP requires activation of downstream eNOS signaling. To our knowledge, this is the first report showing functional impairment of TRPV4-eNOS signaling in glaucomatous primary human TM cells. In POAG, TRPV4-eNOS impairment may perhaps be affecting the TM cell’s ability to detect and mitigate pathophysiological changes in the AH outflow pathway. From a clinical perspective, this study identifies a key physiological pathway responsible for homeostatic regulation of IOP in normal human beings, which is impaired in glaucoma patients. Future studies will target this pathway in order to alleviate disease-associated pathology and restore normal IOP.

## Methods and Materials

### Antibodies and Reagents.

Rabbit TRPV4 antibody (1:250; catalog number ACC034; Alomone Labs), rabbit phosphorylated-eNOS (p-eNOS) antibody (1:1,000; catalog number PA5-35879; Thermo Fisher Scientific), rabbit total eNOS antibody (1:100 to 500; catalog number NB300-500; Novus Biologicals), rabbit α-SMA antibody (1:100; catalog number ab5694; Abcam), GSK1016790A (catalog number G0798-10MG; Sigma Aldrich), GSK2193874 (catalog number 5106; Tocris), NONOate (catalog number 136587–13-8; Cayman Chemicals), and pan-NOS inhibitor l-NAME (N5751-5G; Sigma Aldrich) were used. Primary antibodies for TRPV4 and eNOS (total and phosphorylated) were validated using ocular tissues from TRPV4 and eNOS knockout mice (*SI Appendix*, Fig. S8). In addition, the same TRPV4 antibody was used in our previous study (54).

### Animal Models.

Male and female C57BL/6J and NOS3^−/−^ mice on pure C57BL/6J background were obtained from the Jackson Laboratory. The generation of TRPV4^f/f^ mice on pure C57BL/6J background has been described previously (55). All mice were 3 to 4 mo old at the start of experiments. Animal studies and care were performed in compliance with the Association for Research in Vision and Ophthalmology Statement for the Use of Animals in Ophthalmic and Vision Research and the University of North Texas Health Science Center Institutional Animal Care and Use Committee regulations (approved protocol: IACUC2018-0032). Mice were housed under controlled temperature (21 to 26 °C) and humidity (40 to 70%), with a 12 h light/12 h dark cycle (8:00 PM to 8:00 AM). Food and water were provided ad libitum. The number of animals used in each experiment is indicated in the corresponding figure legends.

### IOP Measurement.

Nighttime IOP measurements were performed using Tonolab rebound tonometry (Colonial Medical Supply) in isoflurane anesthetized (2.5% isoflurane and 0.8 L/minute O_2_) mice as previously described (56–59). Nighttime IOPs were measured under low-intensity red light during nighttime in dark-adapted conditions. An average of five individual readings were considered as one reading, and six of such readings were recorded for each eye. All IOP measurements were performed in a masked manner.

### AH Outflow Facility Measurement.

AH outflow facility (C) was measured in live anesthetized mice using constant-flow infusion method in a masked manner as described previously (27, 60–62) and in *SI Appendix*.

### Intravitreal Injection for Viral Vectors.

A 33-gauge needle with a glass microsyringe (10 µL volume; Hamilton Company) was used for intravitreal injection of Ad5-Cre and Ad5-Empty. The eye was proptosed, and the needle was inserted through the equatorial sclera into the vitreous chamber at an angle of ∼45°, carefully avoiding contact with the posterior lens capsule or the retina. Viral vectors in a volume of 2 µL (1 × 10^7^ particle forming units [pfu]/eye) were slowly injected into the vitreous, and the needle was then left in place for an additional 30 s to avoid leakage before being withdrawn.

### Cell Culture.

Primary human TM cells were isolated and characterized as reported previously (63, 64). Human donor information was deidentified prior to use in this study. Primary human TM cells from normal (*n* = 7 cell strains) and glaucoma (*n* = 7 cell strains) donor eyes and transformed GTM3 cells were cultured in Dulbecco’s Modified Eagle’s Medium (DMEM; Sigma Aldrich; catalog D6046-500ML), supplemented with 10% fetal bovine serum (FBS), 1% penicillin–streptomycin (Pen-Strep) (Sigma Aldrich), and l-glutamine and incubated at 5% CO_2_ and 37 °C in humidified conditions, as described previously (58, 59, 65–67). Cell lysates were collected in 1× radioimmunoprecipitation assay buffer (Sigma Aldrich; catalog R0278) containing a protease inhibitor mixture and phosphatase inhibitor (Roche Life Sciences). Primary human SC cells (*n* = 3 strain) were obtained from Dr. Ellis laboratory and are characterized and cultured as reported previously (68). The detailed information of primary human TM and SC cells, including age, race, sex, passage, and pathological conditions of donor eyes, is summarized in Table 1.

**Table 1.** Donor information for human trabecular meshwork and Schlemm’s Canal cells used in the study

Primary TM cell line	Age	Sex	Race	Passages	Ocular disease(s)
NTM 2252 OS	73	M	NS	3–6	None
NTM G1	66	F	African American	5–8	None
NTM 1848 OD	43	M	African American	3–7	None
NTM 17–3431	55	F	Caucasian	5–8	None
NTM 8467	56	M	Caucasian	5–8	None
NTM 895–03	63	F	NS	7–9	None
NTM 8055	53	M	Caucasian	3–7	None
GTM 1096	99	M	Caucasian	4–7	Glaucoma
GTM 1480 OD	70	M	Caucasian	4–8	Glaucoma
GTM 1480 OS	70	M	Caucasian	4–8	Glaucoma
GTM 3817 OD	68	F	Caucasian	3–6	Glaucoma
GTM 3817 OS	68	F	Caucasian	3–6	Glaucoma
GTM 28462	74	M	Caucasian	4–6	Glaucoma
GTM 7600	96	F	Caucasian	4–7	Glaucoma
Primary SC cell line					
SC 14	14	F	Caucasian	3–6	None
SC 24	24	M	Caucasian	3–6	None
SC 48	48	F	Caucasian	3–6	None

F, female; M, male; NS, not specified.

### Ex Vivo Culture of Human Corneoscleral Segments.

Transplant ineligible, deidentified human corneoscleral segments were acquired from Lions Eye Institute in conformity with the guidelines outlined in the Declaration of Helsinki. According to approved protocols for the Lions Eye Institute, informed consent was obtained from the next of kin for use of ocular tissues for research purposes. Upon receipt, the corneoscleral segments were washed three times with phosphate-buffered saline (PBS) (7.4 pH; Sigma Aldrich; catalog 806552) and then cultured at 37 °C and 5% CO_2_ in phenol red–free DMEM (Sigma Aldrich; catalog number D4947-500ML), supplemented with 10% FBS (Sigma Aldrich), l-glutamine (Sigma Aldrich), and 1% Pen-Strep (Sigma Aldrich) as described previously (35). The corneoscleral segments are dissected within 12 to 24 h of death and preserved in media by Lions Eye Institute. The corneoscleral segments preserved in media were received within 2 to 5 d postmortem. We have previously shown that corneoscleral segments preserved in the media maintain TM viability (35, 69). The corneoscleral segments were utilized within 24 h after receiving.

### DAF-FM Assay for NO Detection.

DAF-FM (Sigma Aldrich) assay was performed to detect NO, as described previously (69), with minor modifications. Human donor corneoscleral segments were divided into quadrants and cultured in phenol red–free DMEM (Sigma Aldrich), supplemented with 0.2% FBS and 1% Pen-Strep (Sigma Aldrich). The control and experimental treatments were performed on the quadrants of the same eye to reduce variability associated with the use of contralateral eye. Quadrants were pretreated with 10 µM DAF-FM (Sigma Aldrich) dye and incubated at 37 °C for 30 min. Following incubation, quadrants were washed three times with PBS and incubated for an additional 30 min at 37 °C to allow proper incorporation and activation of intracellular dye. The quadrants were then treated either with vehicle (0.001% DMSO), GSK101 (20 nM), l-NAME (100 µM; negative control), or NONOate (100 µM; positive control) and further incubated for 30 min at 37 °C. After the incubation period, the quadrants were washed three times with PBS and prepared for imaging. The TM rim (including the inner wall of SC) was carefully dissected from the unfixed corneoscleral quadrants and placed between coverslips for imaging under the fluorescence microscope (Keyence).

Primary human TM cells were cultured on glass bottom 24-well plates in phenol red–free DMEM (Sigma Aldrich), supplemented with 0.2% FBS and 1% Pen-Strep (Sigma Aldrich). Cells were pretreated with 10 µM DAF-FM (Sigma Aldrich) dye and incubated at 37 °C for 30 min. Following incubation, cells were washed three times with PBS and incubated for an additional 30 min at 37 °C. The cells were then treated either with vehicle (0.001% DMSO), GSK101 (20 nM), l-NAME (100 μM), or NONOate (100 μM) and further incubated for 30 min at 37 °C. After the incubation period, the cells were washed three times with PBS and imaged under a fluorescence microscope (Keyence). DAF-FM fluorescence images were analyzed by quantifying fluorescence intensity per unit area (IntDen/µm^2^) using ImageJ (NIH), as described previously (66, 70).

### Immunostaining.

Paraffin-embedded human tissue sections from age-matched normal (*n* = 6) and glaucoma (*n* = 5) donors were immunostained as described in *SI Appendix*.

### Western Blot Analysis.

Total protein (∼30 μg) from cell lysates or corneoscleral segment TM rim lysates were run on denaturing 4 to 12% gradient polyacrylamide ready-made gels (NuPAGE Bis-Tris gels, Life Technologies) and transferred onto polyvinylidene difluoride membranes. Blots were blocked with 10% nonfat milk in PBS + Tween 20 (0.1%) solution (1× PBS + 0.1% Tween 20; Sigma Aldrich) for 2 h and then incubated overnight with specific primary antibodies at 4 °C on a rotating shaker at 100 rpm. The membranes were washed thrice with PBST and incubated with corresponding horseradish peroxidase-conjugated secondary antibody for 2 h. The proteins were then visualized using SuperSignal West Femto Maximum Sensitivity detection reagent (Life Technologies). Densitometric analysis was performed on immunoblots using ImageJ (NIH).

### Fluid Flow/Shear Stress.

Fluid flow/shear stress was applied to normal and glaucomatous primary human TM cells using the Ibidi pump system (Ibidi). Primary TM cells (2 × 10^5^ cells/slide) were seeded onto IbiTreat μ-slides I^0.6^ (Ibidi) and placed in an incubator at 37 °C with 5.0% CO_2_. Primary TM cells were allowed to settle for 3 d and become confluent before induction of shear. The Ibidi pump system (Ibidi) was set up as per the manufacturer’s instructions, and proprietary software was used to control the level of shear applied to cells by controlling total media flow rate. Shear levels were simulated as 0.0, 1.0, and 3.0 dyne/cm^2^ for no-shear, low-shear, and high-shear conditions, respectively. DAF-FM assay (Milllipore Sigma) was performed to determine the levels of NO produced in response to shear.

### Ca^2+^ Imaging and Analysis.

Primary human TM cells from normal individuals or glaucoma patients were incubated with fluo-4 AM (10 μM) and pluronic acid (0.04%) at 30 °C for 30 min. Ca^2+^ images were acquired at 30 frames per second using Andor Revolution WD (with Borealis) spinning disk confocal imaging system (Andor Technology) comprising an upright Nikon microscope with a 60× water dipping objective (numerical aperture 1.0) and an electron multiplying charge coupled device camera (71). Fluo-4 was excited using a 488 nm solid-state laser, and emitted fluorescence was captured using a 525/36 nm band-pass filter. TM cells were treated with cyclopiazonic acid (CPA; 20 µM), a sarco-endoplasmic reticulum Ca^2+^-ATPase inhibitor, for 10 min. CPA, per se, does not alter the activity of TRPV4 sparklets (54, 72). TRPV4 sparklet activity was recorded 5 min after the administration of TRPV4 activator GSK1016790A (GSK101, 3 nM). The specific TRPV4 channel inhibitor GSK2193874 (GSK219, 100 nM, 10 min) was used to inhibit TM TRPV4 sparklet activity.

The flow rate of the physiological salt solution was adjusted to obtain a shear stress of 1 dyne/cm^2^, as calculated using the equation

$$Q=\;\frac{\tau .w.h^{2}}{6.\mu },$$

where *Q* is the flow rate, τ is the shear stress, *w* is the width of the flow chamber, *h* is the height of the flow chamber, and *µ* is the viscosity of the solution (0.9 cP).

Ca^2+^ images were analyzed using a custom-designed SparkAn software (developed by Dr. Adrian Bonev, University of Vermont) (23, 54, 72). F/F_0_ traces were obtained by placing a 1.7 μm^2^ ROI at the peak event amplitude and were filtered using a Gaussian filter and a cutoff corner frequency of 4 Hz. TRPV4 Ca^2+^ sparklet activity was analyzed as previously described (54, 73). TRPV4 sparklet activity is calculated as NP_O_ (where N is number of TRPV4 channels per site, and P_O_ is the open state probability of the channel), which was calculated using the Single-Channel Search module of Clampfit, previously reported quantal amplitudes derived from all-points histograms (0.29 ΔF/F_0_ for fluo-4-loaded mesenteric arteries), and the following equation (13):

$$NPo=\left[\frac{\left(T_{level1}+2T_{level2}+3T_{level3}+4T_{level4}\right)}{T_{total}}\right],$$

where *T* represents the dwell time at each quantal level, and *T*_*total*_ is the total recording duration. TRPV4 sparklet sites per cell were obtained by dividing total sparklet sites in a field by the number of TM cells in that field.

### TM Cell Patch Clamp.

TRPV4 channel currents were recorded in primary human TM cells. Patch clamp electrodes (pipette resistance ∼4 to 6 ΩM) were pulled from borosilicate glass (outer diameter: 1.5 mm; inner diameter: 1.17 mm; Sutter Instruments, Novato) using Narishige PC-100 puller (Narishige International USA, Inc.) and polished using MicroForge MF-830 polisher (Narishige International USA, Inc.). Whole-cell currents were measured at room temperature using a conventional whole-cell patch configuration. The bathing solution consisted of 10 mM Hepes, 134 mM NaCl, 6 mM KCl, 2 mM CaCl_2_, 10 mM glucose, and 1 mM MgCl_2_ (adjusted to pH 7.4 with NaOH). The intracellular solution consisted of 20 mM CsCl, 100 mM Cs-aspartate, 1 mM MgCl_2_, 4 mM ATP, 0.08 mM CaCl_2_, 10 mM BAPTA (1,2-bis(o-aminophenoxy)ethane-N,N,N′,N′-tetraacetic acid), and 10 mM Hepes, pH 7.2 (adjusted with CsOH). Experiments were performed in the presence of ruthenium red (RuR, 1 μM) in the bathing solution, as described previously (13). RuR, a polyvalent cation, exhibits a voltage-dependent block of TRPV channels and is driven out of the pore by membrane depolarization. With RuR present, GSK101 at 10 nM had little effect on membrane currents at −50 mV. Depolarizing voltage ramps from −100 mV to +100 mV (200 ms) were applied to unblock TRPV4 channels. TRPV4 currents were measured at +100 mV, a voltage close to the Ca^2+^ equilibrium potential. GSK101 (10 nM)-induced outward current was assessed 5 min after the addition of GSK101. The effect of TRPV4 inhibitor GSK219 (100 nM) on the outward currents was recorded 5 min after the addition of GSK219. The data were acquired using HEKA EPC 10 amplifier and PatchMaster v2 × 90 programs (Harvard Bioscience). The patch clamp recordings were analyzed using FitMaster v2 × 73.2 (Harvard Bioscience) and MATLAB R2018a (MathWorks).

### Statistical Analysis.

Statistical analysis was performed using GraphPad Prism 8. Data are expressed in means ± SEM. Two-group comparisons were analyzed by unpaired Student’s *t* test. Multiple comparisons were analyzed by two-way ANOVA with Bonferroni post hoc test.

## Supplementary Material

Supplementary File

Supplementary File

Supplementary File

## Data Availability

All data are included in the article and/or supporting information.

## References

[1] H. A. Quigley, A. T. Broman, The number of people with glaucoma worldwide in 2010 and 2020. Br. J. Ophthalmol. 90, 262–267 (2006). 16488940 10.1136/bjo.2005.081224 PMC1856963

[2] Y. C. Tham., Global prevalence of glaucoma and projections of glaucoma burden through 2040: A systematic review and meta-analysis. Ophthalmology 121, 2081–2090 (2014). 24974815 10.1016/j.ophtha.2014.05.013

[3] H. A. Quigley, Neuronal death in glaucoma. Prog. Retin. Eye Res. 18, 39–57 (1999). 9920498 10.1016/s1350-9462(98)00014-7

[4] J. Rosenthal, M. C. Leske, Open-angle glaucoma risk factors applied to clinical area. J. Am. Optom. Assoc. 51, 1017–1024 (1980). 7440868

[5] E. R. Tamm, B. M. Braunger, R. Fuchshofer, Intraocular pressure and the mechanisms involved in resistance of the aqueous humor flow in the trabecular meshwork outflow pathways. Prog. Mol. Biol. Transl. Sci. 134, 301–314 (2015). 26310162 10.1016/bs.pmbts.2015.06.007

[6] T. S. Acott., Intraocular pressure homeostasis: Maintaining balance in a high-pressure environment. J. Ocul. Pharmacol. Ther. 30, 94–101 (2014). 24401029 10.1089/jop.2013.0185 PMC3991985

[7] K. E. Keller, M. Aga, J. M. Bradley, M. J. Kelley, T. S. Acott, Extracellular matrix turnover and outflow resistance. Exp. Eye Res. 88, 676–682 (2009). 19087875 10.1016/j.exer.2008.11.023 PMC2700052

[8] B. M. Braunger, R. Fuchshofer, E. R. Tamm, The aqueous humor outflow pathways in glaucoma: A unifying concept of disease mechanisms and causative treatment. Eur. J. Pharm. Biopharm. 95, 173–181 (2015). 25957840 10.1016/j.ejpb.2015.04.029

[9] W. G. Darby., Shear stress sensitizes TRPV4 in endothelium-dependent vasodilatation. Pharmacol. Res. 133, 152–159 (2018). 29787869 10.1016/j.phrs.2018.05.009

[10] S. A. Mendoza., TRPV4-mediated endothelial Ca2+ influx and vasodilation in response to shear stress. Am. J. Physiol. Heart Circ. Physiol. 298, H466–H476 (2010). 19966050 10.1152/ajpheart.00854.2009 PMC2822567

[11] R. Kohler, J. Hoyer, “Role of TRPV4 in the mechanotransduction of shear stress in endothelial cells” in TRP Ion Channel Function in Sensory Transduction and Cellular Signaling Cascades, W. B. Liedtke, S. Heller, Eds. (CRC Press/Taylor & Francis, Boca Raton, FL, 2007). 21204486

[12] V. Hartmannsgruber., Arterial response to shear stress critically depends on endothelial TRPV4 expression. PLoS One 2, e827 (2007). 17786199 10.1371/journal.pone.0000827 PMC1959246

[13] S. K. Sonkusare., Elementary Ca2+ signals through endothelial TRPV4 channels regulate vascular function. Science 336, 597–601 (2012). 22556255 10.1126/science.1216283 PMC3715993

[14] J. P. White., TRPV4: Molecular conductor of a diverse orchestra. Physiol. Rev. 96, 911–973 (2016). 27252279 10.1152/physrev.00016.2015

[15] N. Luo., Primary cilia signaling mediates intraocular pressure sensation. Proc. Natl. Acad. Sci. U.S.A. 111, 12871–12876 (2014). 25143588 10.1073/pnas.1323292111 PMC4156748

[16] D. A. Ryskamp., TRPV4 regulates calcium homeostasis, cytoskeletal remodeling, conventional outflow and intraocular pressure in the mammalian eye. Sci. Rep. 6, 30583 (2016). 27510430 10.1038/srep30583 PMC4980693

[17] R. Fernández-Durango., Expression of nitrotyrosine and oxidative consequences in the trabecular meshwork of patients with primary open-angle glaucoma. Invest. Ophthalmol. Vis. Sci. 49, 2506–2511 (2008). 18296660 10.1167/iovs.07-1363

[18] W. D. Stamer, Y. Lei, A. Boussommier-Calleja, D. R. Overby, C. R. Ethier, eNOS, a pressure-dependent regulator of intraocular pressure. Invest. Ophthalmol. Vis. Sci. 52, 9438–9444 (2011). 22039240 10.1167/iovs.11-7839 PMC3293415

[19] J. Y. Chang., Role of nitric oxide in murine conventional outflow physiology. Am. J. Physiol. Cell Physiol. 309, C205–C214 (2015). 26040898 10.1152/ajpcell.00347.2014 PMC4537932

[20] W. M. Dismuke, C. C. Mbadugha, D. Z. Ellis, NO-induced regulation of human trabecular meshwork cell volume and aqueous humor outflow facility involve the BKCa ion channel. Am. J. Physiol. Cell Physiol. 294, C1378–C1386 (2008). 18385281 10.1152/ajpcell.00363.2007

[21] H. Kotikoski., Comparison of nitric oxide donors in lowering intraocular pressure in rabbits: Role of cyclic GMP. J. Ocul. Pharmacol. Ther. 18, 11–23 (2002). 11858611 10.1089/108076802317233171

[22] G. W. Heyne, J. A. Kiland, P. L. Kaufman, B. T. Gabelt, Effect of nitric oxide on anterior segment physiology in monkeys. Invest. Ophthalmol. Vis. Sci. 54, 5103–5110 (2013). 23800771 10.1167/iovs.12-11491 PMC3729238

[23] C. Marziano., Nitric oxide-dependent feedback loop regulates transient receptor potential vanilloid 4 (TRPV4) channel cooperativity and endothelial function in small pulmonary arteries. J. Am. Heart Assoc. 6, e007157 (2017). 29275372 10.1161/JAHA.117.007157 PMC5779028

[24] D. C. Hill-Eubanks, A. L. Gonzales, S. K. Sonkusare, M. T. Nelson, Vascular TRP channels: Performing under pressure and going with the flow. Physiology (Bethesda) 29, 343–360 (2014). 25180264 10.1152/physiol.00009.2014 PMC4214829

[25] P. D. Cabral, J. L. Garvin, TRPV4 activation mediates flow-induced nitric oxide production in the rat thick ascending limb. Am. J. Physiol. Renal Physiol. 307, F666–F672 (2014). 24966090 10.1152/ajprenal.00619.2013 PMC4166729

[26] S. V. Sukumaran., TRPV4 channel activation leads to endothelium-dependent relaxation mediated by nitric oxide and endothelium-derived hyperpolarizing factor in rat pulmonary artery. Pharmacol. Res. 78, 18–27 (2013). 24075884 10.1016/j.phrs.2013.09.005

[27] J. C. Millar, A. F. Clark, I. H. Pang, Assessment of aqueous humor dynamics in the mouse by a novel method of constant-flow infusion. Invest. Ophthalmol. Vis. Sci. 52, 685–694 (2011). 20861483 10.1167/iovs.10-6069

[28] J. C. Millar, I. H. Pang, W. H. Wang, Y. Wang, A. F. Clark, Effect of immunomodulation with anti-CD40L antibody on adenoviral-mediated transgene expression in mouse anterior segment. Mol. Vis. 14, 10–19 (2008). 18246028 PMC2267727

[29] I. H. Pang, J. C. Millar, A. F. Clark, Elevation of intraocular pressure in rodents using viral vectors targeting the trabecular meshwork. Exp. Eye Res. 141, 33–41 (2015). 26025608 10.1016/j.exer.2015.04.003 PMC4628881

[30] R. B. Kasetti., Autophagy stimulation reduces ocular hypertension in murine glaucoma model via autophagic degradation of mutant myocilin. JCI Insight 6, 143359 (2021). 33539326 10.1172/jci.insight.143359 PMC8021112

[31] Y. A. Nikolaev., Mammalian TRP ion channels are insensitive to membrane stretch. J. Cell Sci. 132, jcs238360 (2019). 31722978 10.1242/jcs.238360 PMC6918743

[32] S. Baratchi., Shear stress mediates exocytosis of functional TRPV4 channels in endothelial cells. Cell. Mol. Life Sci. 73, 649–666 (2016). 26289129 10.1007/s00018-015-2018-8 PMC11108432

[33] J. Aliancy, W. D. Stamer, B. Wirostko, A review of nitric oxide for the treatment of glaucomatous disease. Ophthalmol. Ther. 6, 221–232 (2017). 28584936 10.1007/s40123-017-0094-6 PMC5693832

[34] N. E. Ashpole, D. R. Overby, C. R. Ethier, W. D. Stamer, Shear stress-triggered nitric oxide release from Schlemm’s canal cells. Invest. Ophthalmol. Vis. Sci. 55, 8067–8076 (2014). 25395486 10.1167/iovs.14-14722 PMC4266075

[35] R. B. Kasetti, P. D. Patel, P. Maddineni, G. S. Zode, Ex-vivo cultured human corneoscleral segment model to study the effects of glaucoma factors on trabecular meshwork. PLoS One 15, e0232111 (2020). 32579557 10.1371/journal.pone.0232111 PMC7314024

[36] J. A. Nathanson, M. McKee, Identification of an extensive system of nitric oxide-producing cells in the ciliary muscle and outflow pathway of the human eye. Invest. Ophthalmol. Vis. Sci. 36, 1765–1773 (1995). 7543462

[37] A. Schneemann, B. G. Dijkstra, T. J. van den Berg, W. Kamphuis, P. F. Hoyng, Nitric oxide/guanylate cyclase pathways and flow in anterior segment perfusion. Graefes Arch. Clin. Exp. Ophthalmol. 240, 936–941 (2002). 12486517 10.1007/s00417-002-0559-7

[38] S. Doganay, C. Evereklioglu, Y. Turkoz, H. Er, Decreased nitric oxide production in primary open-angle glaucoma. Eur. J. Ophthalmol. 12, 44–48 (2002). 11936443 10.1177/112067210201200109

[39] J. M. Bradley., Effect of matrix metalloproteinases activity on outflow in perfused human organ culture. Invest. Ophthalmol. Vis. Sci. 39, 2649–2658 (1998). 9856774

[40] M. A. Johnstone, Intraocular pressure regulation: Findings of pulse-dependent trabecular meshwork motion lead to unifying concepts of intraocular pressure homeostasis. J. Ocul. Pharmacol. Ther. 30, 88–93 (2014). 24359130 10.1089/jop.2013.0224 PMC3991971

[41] D. WuDunn, Mechanobiology of trabecular meshwork cells. Exp. Eye Res. 88, 718–723 (2009). 19071113 10.1016/j.exer.2008.11.008

[42] J. Hirt, P. B. Liton, Autophagy and mechanotransduction in outflow pathway cells. Exp. Eye Res. 158, 146–153 (2017). 27373974 10.1016/j.exer.2016.06.021 PMC5199638

[43] O. Yarishkin., Piezo1 channels mediate trabecular meshwork mechanotransduction and promote aqueous fluid outflow. J. Physiol. 599, 571–592 (2021). 33226641 10.1113/JP281011 PMC7849624

[44] P. P. Prosseda., Optogenetic stimulation of phosphoinositides reveals a critical role of primary cilia in eye pressure regulation. Sci. Adv. 6, eaay8699 (2020). 32494665 10.1126/sciadv.aay8699 PMC7190330

[45] F. Stumpff, M. Wiederholt, Regulation of trabecular meshwork contractility. Ophthalmologica 214, 33–53 (2000). 10657743 10.1159/000027471

[46] N. Toda, M. Nakanishi-Toda, Nitric oxide: Ocular blood flow, glaucoma, and diabetic retinopathy. Prog. Retin. Eye Res. 26, 205–238 (2007). 17337232 10.1016/j.preteyeres.2007.01.004

[47] L. K. Wareham, E. S. Buys, R. M. Sappington, The nitric oxide-guanylate cyclase pathway and glaucoma. Nitric Oxide 77, 75–87 (2018). 29723581 10.1016/j.niox.2018.04.010 PMC6424573

[48] W. M. Dismuke, J. Liang, D. R. Overby, W. D. Stamer, Concentration-related effects of nitric oxide and endothelin-1 on human trabecular meshwork cell contractility. Exp. Eye Res. 120, 28–35 (2014). 24374036 10.1016/j.exer.2013.12.012 PMC3943640

[49] A. Cantó, T. Olivar, F. J. Romero, M. Miranda, Nitrosative stress in retinal pathologies: Review. Antioxidants 8, 543 (2019). 10.3390/antiox8110543 PMC6912788 31717957

[50] R. F. Brubaker, The effect of intraocular pressure on conventional outflow resistance in the enucleated human eye. Invest. Ophthalmol. 14, 286–292 (1975). 1123284

[51] D. L. Epstein, J. W. Rohen, Morphology of the trabecular meshwork and inner-wall endothelium after cationized ferritin perfusion in the monkey eye. Invest. Ophthalmol. Vis. Sci. 32, 160–171 (1991). 1987099

[52] G. Patel., Molecular taxonomy of human ocular outflow tissues defined by single-cell transcriptomics. Proc. Natl. Acad. Sci. U.S.A. 117, 12856–12867 (2020). 32439707 10.1073/pnas.2001896117 PMC7293718

[53] F. Galassi., Nitric oxide proxies and ocular perfusion pressure in primary open angle glaucoma. Br. J. Ophthalmol. 88, 757–760 (2004). 15148207 10.1136/bjo.2003.028357 PMC1772173

[54] M. Ottolini., Local peroxynitrite impairs endothelial transient receptor potential vanilloid 4 channels and elevates blood pressure in obesity. Circulation 141, 1318–1333 (2020). 32008372 10.1161/CIRCULATIONAHA.119.043385 PMC7195859

[55] C. Moore., UVB radiation generates sunburn pain and affects skin by activating epidermal TRPV4 ion channels and triggering endothelin-1 signaling. Proc. Natl. Acad. Sci. U.S.A. 110, E3225–E3234 (2013).Corrected in: *Proc. Natl. Acad. Sci. U.S.A.* **110**, 15502 (2013). 23929777 10.1073/pnas.1312933110 PMC3752269

[56] G. C. Patel., Dexamethasone-induced ocular hypertension in mice: Effects of myocilin and route of administration. Am. J. Pathol. 187, 713–723 (2017). 28167045 10.1016/j.ajpath.2016.12.003 PMC5397678

[57] W. H. Wang, J. C. Millar, I. H. Pang, M. B. Wax, A. F. Clark, Noninvasive measurement of rodent intraocular pressure with a rebound tonometer. Invest. Ophthalmol. Vis. Sci. 46, 4617–4621 (2005). 16303957 10.1167/iovs.05-0781

[58] R. B. Kasetti., Transforming growth factor β2 (TGFβ2) signaling plays a key role in glucocorticoid-induced ocular hypertension. J. Biol. Chem. 293, 9854–9868 (2018). 29743238 10.1074/jbc.RA118.002540 PMC6016452

[59] G. S. Zode., Ocular-specific ER stress reduction rescues glaucoma in murine glucocorticoid-induced glaucoma. J. Clin. Invest. 124, 1956–1965 (2014). 24691439 10.1172/JCI69774 PMC4001532

[60] J. C. Millar, T. N. Phan, I. H. Pang, A. F. Clark, Strain and age effects on aqueous humor dynamics in the mouse. Invest. Ophthalmol. Vis. Sci. 56, 5764–5776 (2015). 26325415 10.1167/iovs.15-16720

[61] J. C. Millar, T. N. Phan, I. H. Pang, Assessment of aqueous humor dynamics in the rodent by constant flow infusion. Methods Mol. Biol. 1695, 109–120 (2018). 29190023 10.1007/978-1-4939-7407-8_11

[62] P. Maddineni., CNS axonal degeneration and transport deficits at the optic nerve head precede structural and functional loss of retinal ganglion cells in a mouse model of glaucoma. Mol. Neurodegener. 15, 48 (2020). 32854767 10.1186/s13024-020-00400-9 PMC7457267

[63] K. E. Keller., Consensus recommendations for trabecular meshwork cell isolation, characterization and culture. Exp. Eye Res. 171, 164–173 (2018). 29526795 10.1016/j.exer.2018.03.001 PMC6042513

[64] D. W. Stamer, B. C. Roberts, D. L. Epstein, R. R. Allingham, Isolation of primary open-angle glaucomatous trabecular meshwork cells from whole eye tissue. Curr. Eye Res. 20, 347–350 (2000). 10855028

[65] G. S. Zode., Reduction of ER stress via a chemical chaperone prevents disease phenotypes in a mouse model of primary open angle glaucoma. J. Clin. Invest. 125, 3303 (2015). 10.1172/JCI82799 PMC4563763 26237042

[66] J. C. Peters, S. Bhattacharya, A. F. Clark, G. S. Zode, Increased endoplasmic reticulum stress in human glaucomatous trabecular meshwork cells and tissues. Invest. Ophthalmol. Vis. Sci. 56, 3860–3868 (2015). 26066753 10.1167/iovs.14-16220 PMC4468426

[67] R. B. Kasetti, P. Maddineni, J. C. Millar, A. F. Clark, G. S. Zode, Increased synthesis and deposition of extracellular matrix proteins leads to endoplasmic reticulum stress in the trabecular meshwork. Sci. Rep. 7, 14951 (2017). 29097767 10.1038/s41598-017-14938-0 PMC5668243

[68] D. Z. Ellis, N. A. Sharif, W. M. Dismuke, Endogenous regulation of human Schlemm’s canal cell volume by nitric oxide signaling. Invest. Ophthalmol. Vis. Sci. 51, 5817–5824 (2010). 20484594 10.1167/iovs.09-5072

[69] P. D. Patel, R. B. Kasetti, S. K. Sonkusare, G. S. Zode, Technical brief: Direct, real-time electrochemical measurement of nitric oxide in ex vivo cultured human corneoscleral segments. Mol. Vis. 26, 434–444 (2020). 32565671 PMC7300198

[70] W. E. Medina-Ortiz, R. Belmares, S. Neubauer, R. J. Wordinger, A. F. Clark, Cellular fibronectin expression in human trabecular meshwork and induction by transforming growth factor-β2. Invest. Ophthalmol. Vis. Sci. 54, 6779–6788 (2013). 24030464 10.1167/iovs.13-12298 PMC3799562

[71] M. Ottolini., Local peroxynitrite impairs endothelial transient receptor potential vanilloid 4 channels and elevates blood pressure in obesity. Circulation 141, 1318–1333 (2020). 32008372 10.1161/CIRCULATIONAHA.119.043385 PMC7195859

[72] K. Hong., TRPV4 (transient receptor potential vanilloid 4) channel-dependent negative feedback mechanism regulates g_q_ protein-coupled receptor-induced vasoconstriction. Arterioscler. Thromb. Vasc. Biol. 38, 542–554 (2018). 29301784 10.1161/ATVBAHA.117.310038 PMC5823749

[73] S. K. Sonkusare., AKAP150-dependent cooperative TRPV4 channel gating is central to endothelium-dependent vasodilation and is disrupted in hypertension. Sci. Signal. 7, ra66 (2014). 25005230 10.1126/scisignal.2005052 PMC4403000

