# Supplementary material for: Impaired TRPV4-eNOS signaling in trabecular meshwork elevates intraocular pressure in glaucoma
Source: Proc Natl Acad Sci U S A. 2021 Apr 14;118(16):e2022461118. doi: 10.1073/pnas.2022461118 (PMC8072326; doi:10.1073/pnas.2022461118)
Supplement: Supplementary File [file pnas.2022461118.sapp.pdf]

SI Appendix

Impaired TRPV4-eNOS signaling in trabecular meshwork elevates intraocular pressure in glaucoma

Patel et al.,

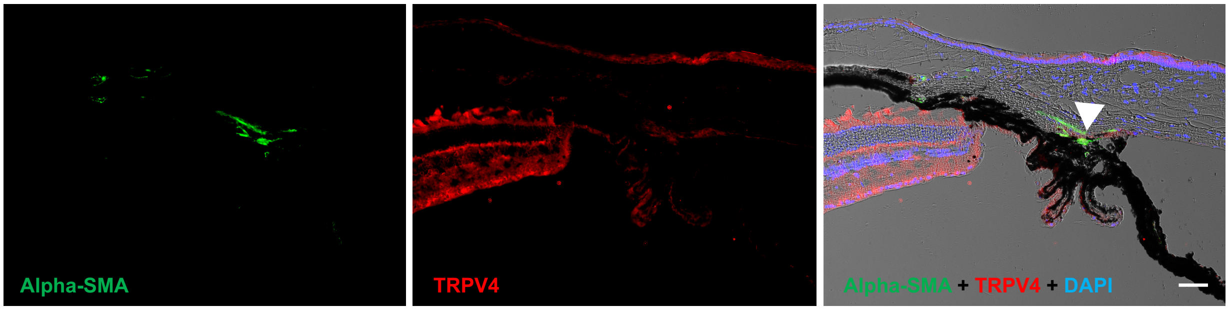

Figure S1. Expression of TRPV4 channels in the mouse TM. Whole eyes from 3-month old C57BL/6J mice were stained with TRPV4 (red) and alpha smooth muscle actin (α-SMA) antibodies. α-SMA was utilized as a marker of TM tissue. TRPV4 (red) was found to be localized to TM region as evident from its co-localization with α-SMA. TRPV4 channel was also found to present in retina and ciliary body. Alpha-SMA (green). n = 5, scale bar = 50

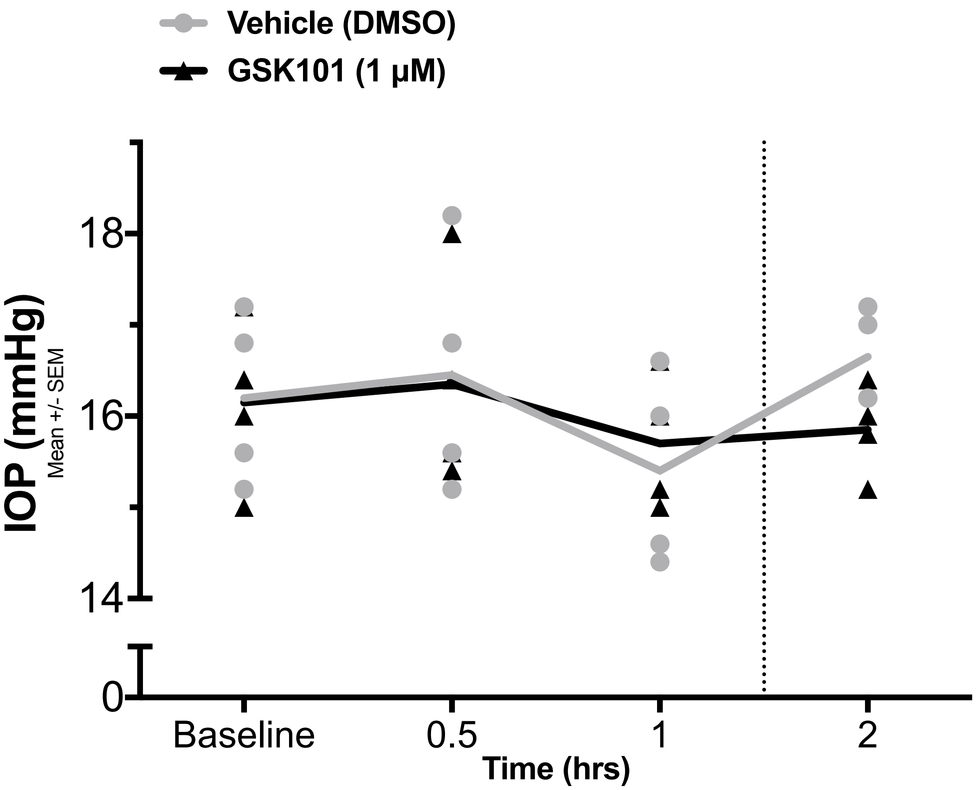

Figure S2: Lower concentration of topical ocular GSK101 does not lower IOP significantly in C57BL/6J mice. 3-months old C57BL/6J mice were given a single topical ocular GSK 101 (1µM) eye drop in one eye and the contralateral eyes were treated with vehicle (DMSO). IOPs were measured before and after treatment. Lower dose of GSK101 did not alter IOP significantly. N=4 mice; 2-way ANOVA.

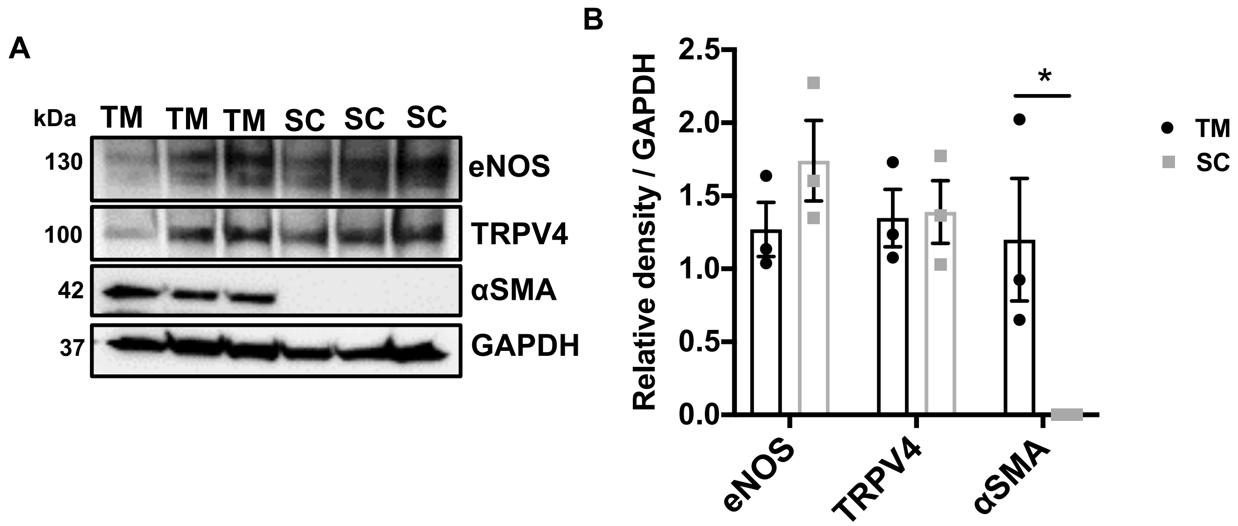

Figure S3. Human primary TM and SC cells were cultured, and cell lysates were subjected to Western blot analysis (**A**) of eNOS, TRPV4 and α-SMA. Both primary TM and SC cells demonstrated similar levels of eNOS and TRPV4 protein. Densitometric analysis (**B**) demonstrated no significant difference in TRPV4 and eNOS levels between TM and SC cells. *$P < 0.05$, n = 3 donor strains/group. α-SMA, a marker of TM cells was only present in TM cells and not in SC cells further validated TM cells.

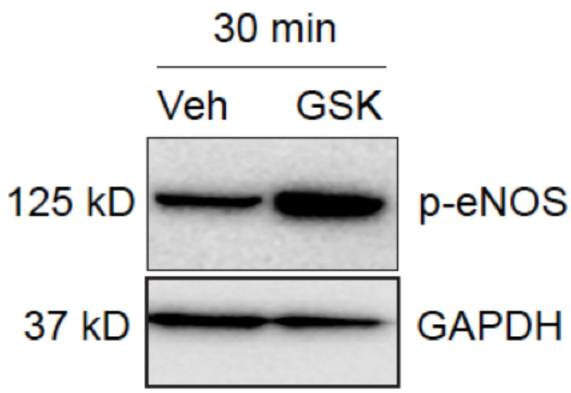

Figure S4. Human primary TM cells were treated with 0.001% DMSO vehicle or 20 nM GSK101 for 30 minutes and total cellular lysates were subjected to Western blot analysis of phosphorylated eNOS. GSK101 induced phosphorylation of eNOS in primary TM cells**.** $P < 0.05$ vs vehicle, n = 3 donors/group.

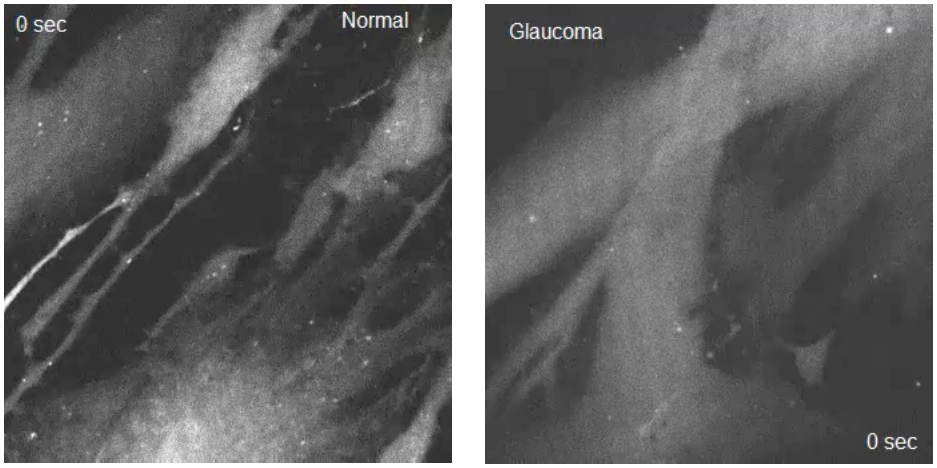

Normal TM cells **Glaucoma TM cells**

Figure S5. Representative videos showing real-time TRPV4 sparklet activity in normal and glaucomatous primary human TM cells. Normal and glaucomatous cells were treated with cyclopiazonic acid (SERCAA inhibitor, CPA, 20 uM) and GSK1016790A (GSK101, TRPV4 activator, 3 nM). Arrowheads represent sites of TRPV4 sparklet activity in intercellular tube-like structures resembling tunneling nanotubes.

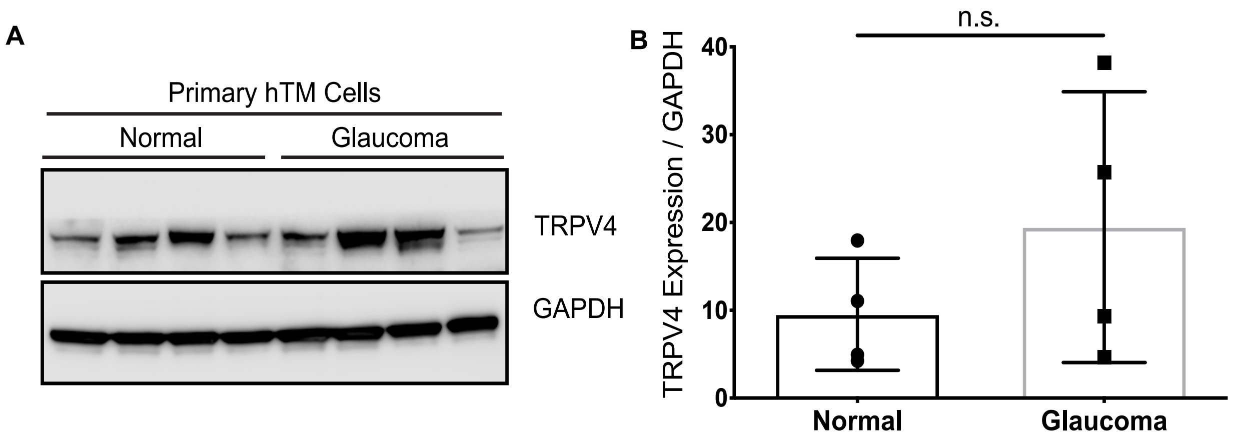

Figure S6. Cellular lysates from primary human TM cells cultured from normal and glaucoma donor eyes were subjected to Western blot analysis of TRPV4 (**A**). Densitometric analysis (**B**) demonstrated that there is no significant change in TRPV4 expression between normal and glaucomatous TM cells. n = 4/group; Unpaired two-tailed t-test.

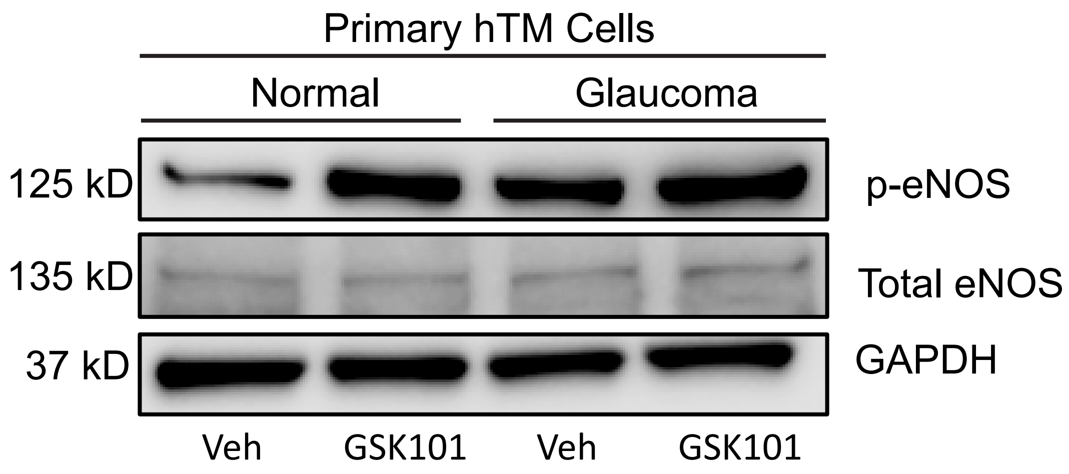

Figure S7. Human primary TM cells cultured from normal and glaucoma donor eyes were treated with GSK101 for 30 minutes and cellular lysates were subjected to western blot analysis of p-eNOS, total eNOS and GAPDH. Representative Western blot showing change in p-eNOS levels in normal and glaucomatous primary human TM cells after treatment with GSK101 or vehicle control. n = 3 cell strains/group.

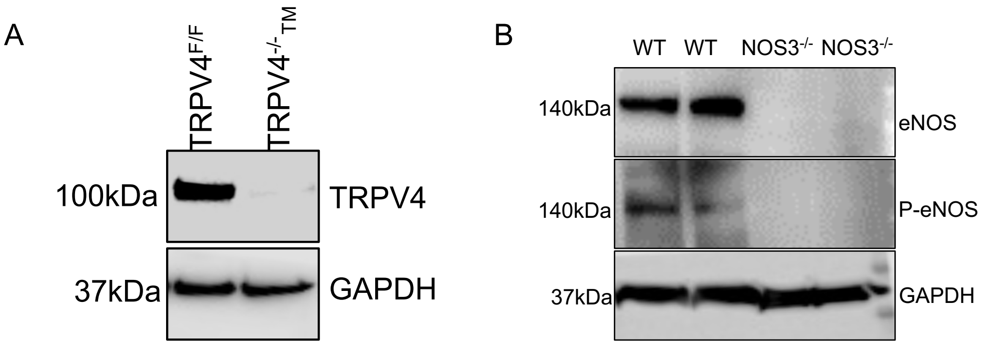

Figure S8: Validation of TRPV4 and eNOS antibodies in mouse ocular tissues. A) $TRPV4^{F/F}$ mice were intracamerally injected with recombinant cre protein in one eye ($TRPV4^{-/-}_{TM}$) and the contralateral eye was injected with PBS. 2-weeks later, anterior segment tissue lysates were subjected to Western blot analysis using TRPV4 antibody. Absence of band in $TRPV4^{-/-}_{TM}$ samples validate the specificity of TRPV4 antibody. **B)** Western blot analysis of total ocular tissue lysates from WT and eNOS knockout mice (n=2) demonstrated absence of total or phosphorylated eNOS in $NOS3^{-/-}$ mice further confirming the specificity of these antibodies.

Methods:

Aqueous humor outflow facility measurement

Animals were anesthetized by intraperitoneal injections of 100/10 mg/kg ketamine/xylazine cocktail. A quarter to half of this dose was administered for maintenance of anesthesia as necessary. Both eyes were then administered a drop of proparacaine HCl (0.5%; Akorn Inc.) for topical anesthesia. Anesthetized mice were placed on a heated mat (37 °C) for maintenance of body temperature throughout the procedure. The anterior chambers of both eyes were cannulated (1-2 mm posterior to the limbus) using a 30-gauge needle inserted across the chamber carefully avoiding contact with the iris, lens capsule or epithelium. The cannula is connected to a calibrated BLPR-2 flow-through blood pressure transducer (World Precision Instruments; WPI) for the continuous determination of pressure within the eye. A topical ocular drop of sterile PBS (Amresco®, Solon) was also instilled upon each eye to prevent corneal drying. The opposing end of each transducer was connected to a 3-way valve, which in turn was connected to a 50 µL glass microsyringe (Hamilton Company) filled with sterile PBS (previously filtered through a 0.2 µm HT Tuffryn Membrane Acrodisc syringe filter; PALL Gelman Laboratory) loaded into an SP101i microdialysis infusion pump (WPI), and (2) an open-ended, variable-height manometer. Signals from the pressure transducers were passed via a TBM4M Bridge Amplifier (WPI) and a Lab-Trax analog-to-digital converter (WPI) to a computer. Data were recorded using Lab-Scribe2 software (WPI). Eyes were initially infused at a constant flow rate of 0.1 µL/minute. Following stabilization of pressure after 10-30 minutes, pressure measurements were recorded over a 15-minute period, then the flow rates were increased sequentially to 0.2, 0.3, 0.4, and 0.5 µL/minute. For each flow rate, three stabilized pressures at 5-minute interval were recorded. C in each

eye of each animal was calculated as the reciprocal of a slope mean stabilized pressure across the different flow rates.

Immunostaining

Paraffin-embedded human tissue sections from age-matched normal (n=6) and glaucoma (n=5) donors were deparaffinized in xylene and rehydrated twice each with 100%, 95%, 70%, and 50% ethanol for 5 minutes. For antigen retrieval, the tissue sections were incubated in citrate buffer (pH 6.0) at 100 ºC for 15 minutes and then at room temperature for another 30 minutes. Tissue sections were blocked with 10% goat serum made in 1x PBS containing 0.2% Triton-X 100 for 2 hours in a dark and humid chamber. Tissue sections were then washed briefly with PBS and immunolabeled with primary antibody and incubated overnight at 4 ºC. Tissue sections incubated without primary antibody served as a negative control. After the incubation, tissue sections were washed three times with PBS and further incubated for 2 hours at room temperature with the appropriate secondary antibodies (1:500; Alexa goat anti-rabbit 568 or Alexa donkey anti-goat; Thermo Fisher Scientific). Tissue sections were washed with PBS and mounted with mounting medium containing DAPI nuclear stain (Vector Labs, Inc.). Images were captured using a fluorescence microscope (Keyence).
